# Diversity of Endolysin Domain Architectures in Bacteriophages Infecting Bacilli

**DOI:** 10.3390/biom14121586

**Published:** 2024-12-11

**Authors:** Olga N. Koposova, Olesya A. Kazantseva, Andrey M. Shadrin

**Affiliations:** Laboratory of Bacteriophage Biology, G.K. Skryabin Institute of Biochemistry and Physiology of Microorganisms, Pushchino Scientific Center for Biological Research of the Russian Academy of Sciences, Federal Research Center, Prospect Nauki, 5, 142290 Pushchino, Russia; koposova@pbcras.ru (O.N.K.); olesyakazantseva@bk.ru (O.A.K.)

**Keywords:** bacteriophage, endolysin, *Bacillus*, antibiotic resistance, antibacterial compounds, domain architectures, N-acetylmuramoyl-L-alanine amidases, N-acetylglucosaminidases, N-acetylmuramidases, endopeptidases, and lytic transglycosylases

## Abstract

The increasing number of antibiotic-resistant bacterial pathogens is a serious problem in medicine. Endolysins are bacteriolytic enzymes of bacteriophages, and a promising group of enzymes with antibacterial properties. Endolysins of bacteriophages infecting Gram-positive bacteria have a modular domain organization. This feature can be used to design enzymes with new or improved properties by modifying or shuffling individual domains. This work is a detailed analysis 1of the diversity of endolysin domains found in bacteriophages infecting bacilli. During the course of the work, a database of endolysins of such bacteriophages was created, and their domain structures were analyzed using the NCBI database, RASTtk, BLASTp, HHpred, and InterPro programs. A phylogenetic analysis of endolysins was performed using MEGA X. In 438 phage genomes, 454 genes of endolysins were found. In the endolysin sequences found, eight different types of catalytic domains and seven types of cell wall binding domains were identified. The analysis showed that many types of endolysin domains have not yet been characterized experimentally. Studies of the properties of such domains will help to reveal the potential of endolysins for the creation of new antibacterial agents.

## 1. Introduction

The increasing spread of antibiotic resistance among bacteria has been one of the main healthcare concerns in recent decades. The widespread and not always warranted use of antibiotics in industry, agriculture, and medicine has significantly accelerated the emergence of strains resistant to certain antibiotics [1]. Representatives of the World Health Organization (WHO) note that the development of new antibacterial agents cannot keep pace with the spread of antibiotic resistance, risking a major setback in medicine by 2050 [2].

Members of the genus *Bacillus* are widespread in the environment, and some of them, in particular *Bacillus anthracis* and *Bacillus cereus sensu stricto* (s.s.), are capable of causing diseases in humans and animals.

*B. anthracis* is the causative agent of a dangerous zoonotic disease, anthrax, the potential sources of which are scattered over the world in the form of cattle burial grounds and natural foci of infection. In Russia alone, at least 35,000 such locations have been registered [3]. According to the Federal Service for Veterinary and Phytosanitary Surveillance, the Federal Center for Animal Health, as of 2014, 14,244 cattle burial grounds were registered in Russia, but the exact number of anthrax burials among them has not been specified. It was noted that 192 anthrax burial grounds were located in flood areas, which increases the risk of their erosion by water, and subsequently the spread of infection. Additionally, 1109 anthrax burial grounds do not meet the sanitary standards, which pose an epidemiological threat [4]. Outbreaks of anthrax occur regularly throughout the world, usually among people involved in working with animals, meat products, and animal hides [5]. Estimating the incidence of anthrax in humans and animals is complicated by the fact that it is difficult for epidemiological services to ensure full control in remote regions. The number of cases among humans is estimated to be between 20,000 and 100,000, annually [6]. Anthrax can occur in three different forms: cutaneous, inhalation (pulmonary), and gastrointestinal (intestinal). The forms differ in localization, symptoms, and mortality rate. The cutaneous form is characterized by the formation of ulcers on the skin. With timely treatment with antibiotics, the mortality rate is reduced to 1% or less, but without treatment, the mortality rate can reach 20%. The inhalation form at the initial stages is similar to the flu in symptoms. If no treatment is given within 48 h after the first symptoms of bacteremia, the mortality rate can reach 95%. The gastrointestinal form manifests itself as acute inflammation of the gastrointestinal tract, in which the mortality rate varies from 25% to 60% [7]. The data emphasize the importance of a timely diagnosis and treatment, especially in the case of pulmonary and gastrointestinal anthrax, where delay significantly increases the risk of death.

The exact contribution of *B. cereus* s.s. to human mortality is difficult to estimate, since not all countries have developed methods for identifying these microorganisms, and not all keep records of such infections. However, it has been reliably confirmed that *B. cereus* s.s. can cause food poisoning and can also be a causative agent of wound, burn, eye, and systemic infections. Incorrectly prescribed antibiotic treatment can be fatal. Thus, in a 2015 study, nine out of twenty-four patients with bacteremia caused by *B. cereus* s.s. died. One reason for that was the prescription of β-lactam antibiotics, to which *B. cereus* s.s. has natural resistance [8]. In Europe, *B. cereus* s.s. is the third most common bacterial pathogen causing foodborne diseases including cases with a fatal outcome [9]. In recent years, several cases have been reported where *B. cereus* s.s. strains were the causative agents of anthrax-like infections. Such strains are called *B. cereus* biovar *anthracis* and possess plasmids with virulence factors typical of *B. anthracis* such as pBCXO1 (similar to pXO1 of *B. anthracis*) and pBC210. Both plasmids encode toxin proteins as well as genes required for the synthesis of a polysaccharide capsule [10].

One of the promising ways to combat the growth of antibiotic resistance is to discover new antimicrobial substances. One kind of such substances are bacteriophage endolysins. Endolysins are synthesized inside the infected bacterial cell at the final stage of the phage lytic cycle. They destroy the peptidoglycan layer of the cell wall, leading to the release of phage progeny. Endolysins can destroy the peptidoglycan of Gram-positive bacteria when acting from the outside of the cell wall as well as from the inside. As a result of such destruction, the turgor pressure in the cell, directed outward from the cell wall, leads to the protrusion of the cytoplasm into the gaps formed by endolysins, which leads to a disruption of the integrity of the bacterial membrane, and consequently cell lysis. For this reason, phage endolysins are considered potential antibacterial agents for medical or industrial use [11].

Endolysins are classified into five classes based on their mechanism of action as well as the location of the covalent bond they cleave: N-acetylmuramoyl-L-alanine amidases, N-acetylglucosaminidases, N-acetylmuramidases, endopeptidases, and lytic transglycosylases. Endolysins of bacteriophages infecting Gram-positive bacteria usually consist of at least two domains: the N-terminal catalytic domain (enzymatically active domain, EAD), which is responsible for cleavage of the peptide glycan, and the C-terminal cell wall binding domain (CBD), which binds specifically to the cell wall, ensuring enzyme specificity to a particular group of bacteria [12]. Sometimes endolysins have more than two domains, for example, the three-domain endolysin of the bacteriophage PlySK1249 infecting *Streptococcus dysgalactiae* [13]. In each of the five classes of endolysins, there are many types of EADs, and the diversity of CBDs is even wider. These domains can be merged with each other in various combinations, providing a wide variety of endolysins. Understanding the properties of individual domains enables the engineering of modified endolysins with the desired characteristics. However, to date, only a small portion of endolysin domains have been experimentally characterized. Endolysins of *Bacillus*-infecting bacteriophages feature impressive domain diversity, which makes these molecules a great source for designing new endolysins by domain shuffling [14]. As of December 2023, the NCBI database contained 438 whole-genome sequences of *Bacillus*-infecting bacteriophages, however, only 28 endolysins of such phages have been experimentally characterized, which is less than seven percent.

In this work, a systematic analysis of endolysin sequences of *Bacillus*-infecting phages was carried out. The domain composition of the endolysins was established, the endolysins were classified into groups based on EAD types, and phylogenetic trees were constructed for each group. This approach allowed us to identify groups of uncharacterized *Bacillus* phage endolysins and their domains in which to focus researchers in future studies. Such studies will enable the development of promising antibacterial compounds for medical and industrial applications.

## 2. Materials and Methods

### 2.1. Database of Bacillus Phage Genomes

To create the database, the following queries were used when searching the NCBI Nucleotide database: “*Bacillus* phage, complete genome”, “*Bacillus* bacteriophage, complete genome”, and “*Bacillus* virus, complete genome”. The following settings were used to filter the query data: “Species: Viruses”, “Molecule types: genomic DNA/RNA”, “Source databases: INSDC (GenBank)”, “Sequence Type: Nucleotide”. A total of 529 sequences were downloaded (data obtained on 4 September 2023), of which 91 sequences were removed because they were duplicates, genomes of phages infecting other bacterial genera, or bacterial genomes. As a result, 438 genomic sequences of *Bacillus*-infecting phages were selected for further analyses.

### 2.2. Database of Bacillus Phage Endolysins

The selected 438 genomes were re-annotated using RASTtk [15]. The annotation data were then manually analyzed to select genes of putative endolysins. If no endolysin genes were found in a phage genome, additional annotation of all open reading frames was performed using BLASTp and HHpred to identify potential endolysins [16,17].

All amino acid sequences of endolysins selected in the first stage were re-examined to refine their functional annotation using BLASTp and HHpred. For BLASTp analysis, the standard database “non-redundant protein sequences (nr)” was used, with the parameter “organism: “Viruses (taxid:10239)” and the algorithm “PSI-BLAST (Position-Specific Iterated BLAST)”. For HHpred analysis, the following domain databases were used: “Pfam-A_v36”, “COG_KOG_v1.0”, “SCOPe70_2.08”, and “NCBI_conserved_Domain(CD)_v3.19” with default parameters. Protein functions were assigned manually based on the results of the automatic annotation.

To accurately determine the domain composition and boundaries of the detected domains, we compared data obtained with BLASTp and HHpred as well as data from a comparative analysis in InterPro with default parameters [18].

Thus, 454 sequences of putative endolysins were obtained. A database of endolysins was formed that included information about their source bacteriophage (full name and ID number), type of EAD and CBD, and sequence ID (Supplementary Table S1).

### 2.3. Classification of Endolysins by Their EAD Types

A phylogenetic analysis of the 454 putative endolysins was performed using MEGA X v.10.2.6 [19]. Separate clades were observed on the phylogenetic proteomic tree (not shown): endolysins were grouped according to their EAD types, which is consistent with the results obtained by Love M. J. et al. [12]. A comparative phylogenetic analysis was performed for EADs from each clade. For this, the complete amino acid sequences of EADs were aligned in MEGA X v.10.2.6 using the ClustalW algorithm [20]. Based on the alignments, phylogenetic trees were constructed for each EAD type using the maximum likelihood method with the following parameters: bootstrap analysis with 500 replicates, and the Jones–Tailor–Thornton model [21,22]. Tree visualization was performed in iTOL v.6.9.1. [23]. Scientific articles describing each group of endolysins were searched for.

## 3. Results

### 3.1. Enzyme Active Domain Types

A total of 438 *Bacillus*-infecting phage genomes were analyzed. In three genomes, endolysin-encoding genes were not detected. In the remaining 435 genomes, 454 genes encoding endolysins were found, which contained 8 different types of EADs and 7 types of CBDs. The analysis revealed 42 different variants of domain combinations: among them, 75 endolysins contained one domain, 268 contained two domains, 92 contained three domains, and 19 contained four domains.

**Figure 1:**
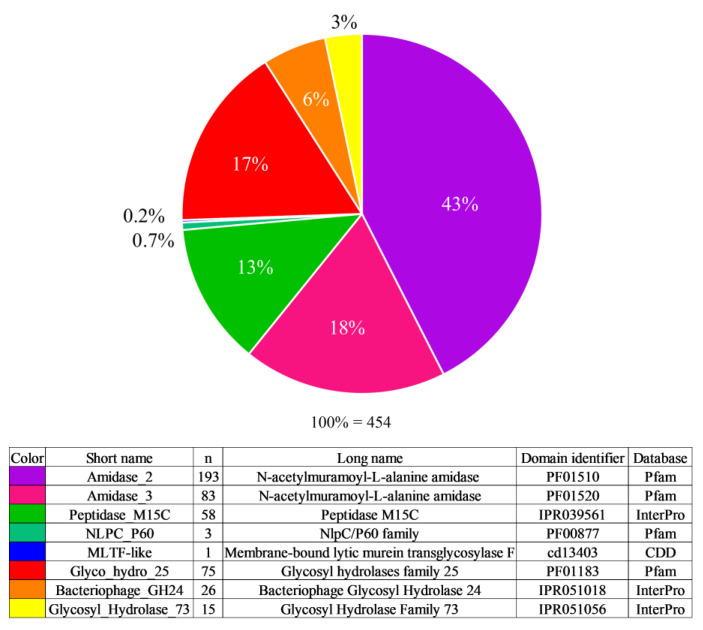
Representation of different types of EADs in the endolysins of *Bacillus*-infecting phages. n—number of endolysins with a domain of the corresponding type.

According to the results of the Pfam, InterPro and CDD database analysis, the most common EAD type found was Amidase_2, which accounted for 43% of the identified catalytic domains. The second common was Amidase_3—18%, then Glyco_hydro_25—17%, Peptidase_M15C—13%, Bacteriophage_GH24—6%, Glycosyl_Hydrolase_73—3%, NLPC_P60—0.7%, and only one endolysin was found that contained an MLTF-like domain—0.2% (Figure 1).

Of the 454 putative endolysins found, only 28 have been experimentally characterized. The most studied were endolysins containing the Amidase_2 domain, of which 13 enzymes were characterized. Six enzymes were characterized that contained the Glyco_hydro_25 domain, five enzymes with the Amidase_3 domain, three enzymes with the Peptidase_M15_3 domain, and only one enzyme with the Glycosyl_Hydrolase_73 domain. No reports on the properties of endolysins with the Bacteriophage_GH24, NLPC_P60, and MLTF-like domains were found.

### 3.2. Cell Wall Binding Domain Types

**Figure 2:**
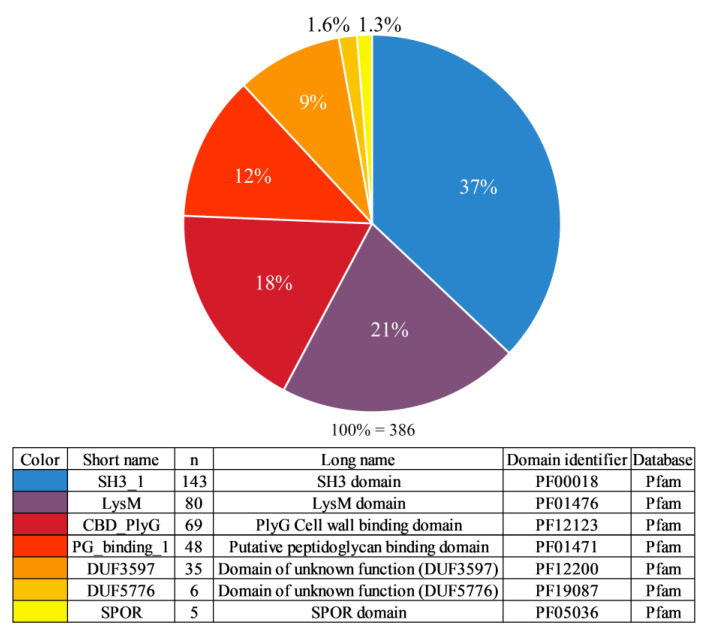
Representation of different types of cell wall binding domains in endolysins of *Bacillus*-infecting phages. n—number of endolysins with a domain of the corresponding type.

The distribution of the CBD types identified was as follows: SH3_1—37%, LysM—21%, CBD_PlyG—18%, PG_binding_1—12%, DUF3597—9%, DUF5776—1.6%, and SPOR—1.3% (Figure 2). The CBD_PlyG domain was previously referred to in a number of publications as the Amidase02_C domain and has been described as a truncated version of the Amidase_2 domain.

The best-studied are endolysins with the SH3 CBD domain—16 such enzymes have been characterized. Ten endolysins have been characterized that contain the CBD_PlyG domain, one with the LysM domain, one with the PG_binding_1 domain, and one with the DUF5776 domain. No publications have been devoted to endolysins with the DUF3597 and SPOR domains.

### 3.3. Phylogenetic Analysis

For endolysins containing the same type of EADs, phylogenetic trees were constructed using the amino acid sequences of their EADs.

**Figure 3:**
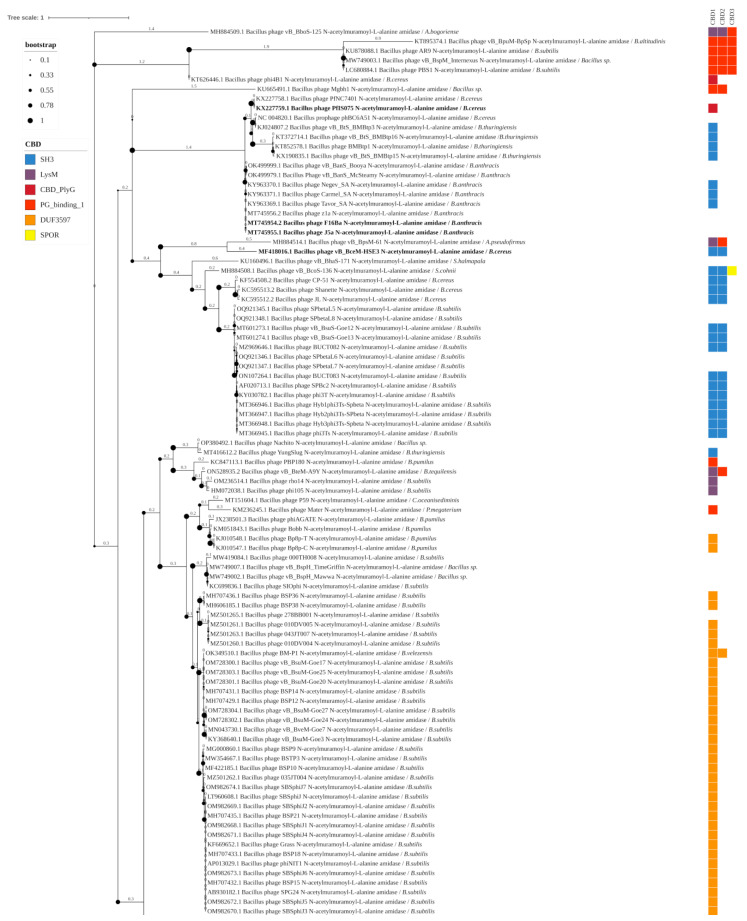

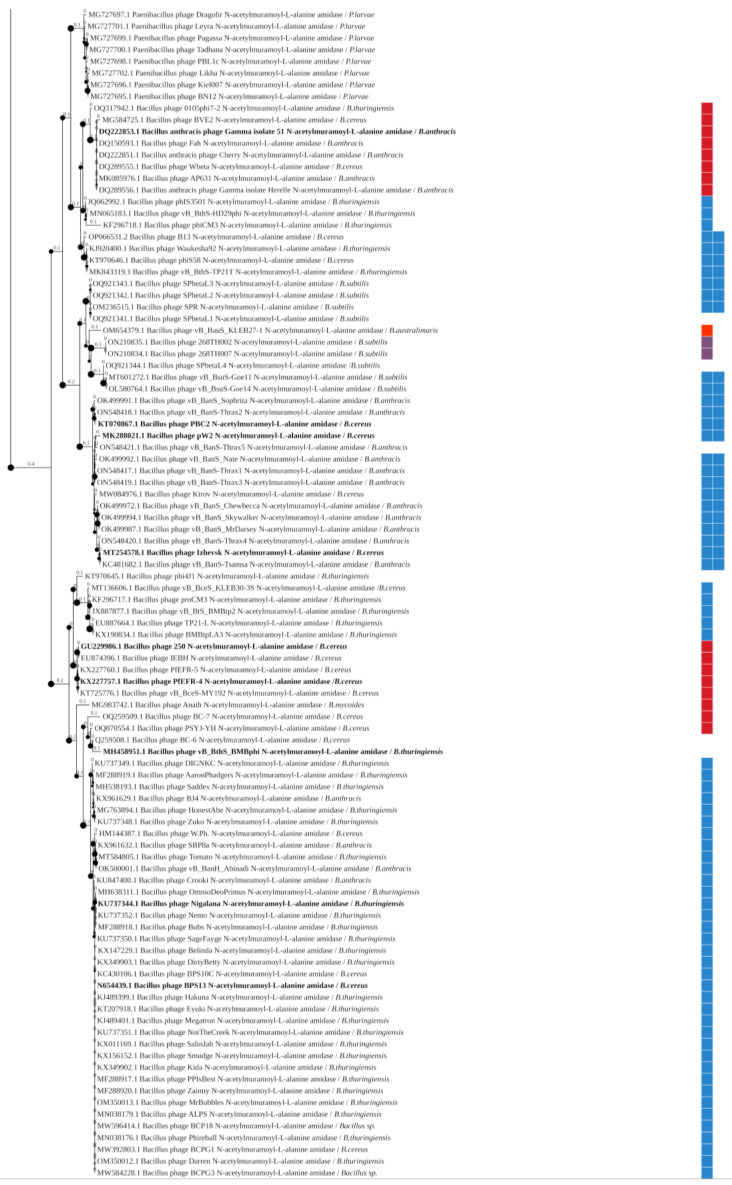
Phylogenetic tree of Amidase_2 domains from the endolysins of *Bacillus*-infecting bacteriophages. The tree was constructed using the maximum likelihood method (bootstrap analysis with 500 replicates) and visualized in iTOL v.6.9.1. Enzymes previously characterized in publications are highlighted in bold. The host bacteria of the bacteriophages, in whose genomes endolysins were identified, are indicated in italics following the “/” symbol.

The Amidase_2 group includes 193 endolysins of *Bacillus*-infecting phages with 6 types of CBDs, forming 12 different combinations of EADs and CBDs. These endolysins include up to three CBDs. Thirteen enzymes of this group have been experimentally characterized (Figure 3). Enzymes containing combinations of several different CBDs were identified: phage vB_BcoS-136 endolysin (EAD: Amidase_2; CBDs: two SH3 domains and one SPOR domain), phage vB_BboS-125 endolysin (EAD: Amidase_2; CBDs: two LysM domains and one PG_binding_1 domain), phage vB_BpsM-61 endolysin (EAD: Amidase_2; CBDs: LysM domain and PG_binding_1 domain), phage vB_BteM-A9Y endolysin (EAD: Amidase_2; CBDs: LysM domain and PG_binding_1 domain), none of them experimentally characterized. Bacterial hosts of phages possessing the Amidase_2 domain-containing endolysins include: *Alkalihalobacterium bogoriense*, *Alkalihalophilus pseudofirmus*, *Bacillus altitudinis*, *Bacillus subtilis*, *Bacillus* sp., *B. cereus* s.s., *B. anthracis*, *Bacillus thuringiensis*, *Sutcliffiella halmapala*, *Sutcliffiella cohnii*, *Bacillus pumilus*, *Bacillus tequilensis*, *Cytobacillus oceanisediminis*, *Priestia megaterium*, *Bacillus velezensis*, *Paenibacillus larvae*, *Bacillus australimaris*, and *Bacillus mycoides*.

**Figure 4:**
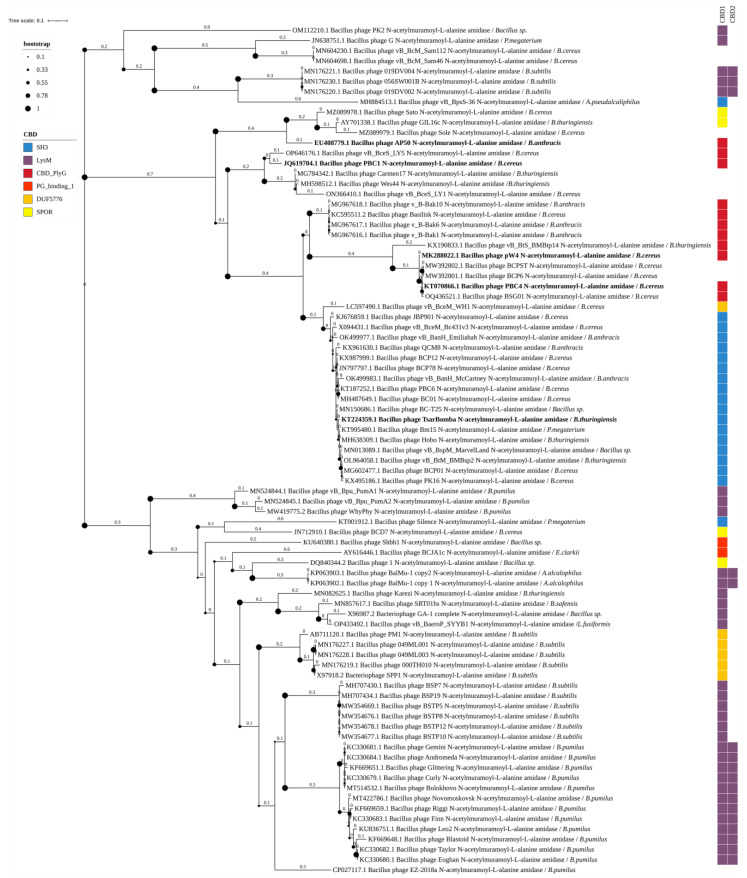
Phylogenetic tree of Amidase_3 domains from the endolysins of *Bacillus*-infecting bacteriophages. The tree was constructed using the maximum likelihood method (bootstrap analysis with 500 replicates) and visualized in iTOL v.6.9.1. Enzymes previously characterized in scientific publications are highlighted in bold. The host bacteria of the bacteriophages, in whose genomes endolysins were identified, are indicated in italics following the “/” symbol.

The Amidase_3 domain group includes 83 out of 454 endolysins of *Bacillus*-infecting phages identified, which have 6 different types of CBDs, forming 7 combinations of domains (Figure 4). Endolysins of this type contain up to two CBDs. These enzymes have not been sufficiently characterized in the scientific literature: only five of them have been described, with four of them containing a combination of the Amidase_3 domain with the CBD_PlyG domain. Bacterial hosts of phages with the Amidase_3 domain include: *Bacillus* sp., *P. megaterium*, *B. cereus* s.s., *B. subtilis*, *Alkalihalobacillus alcalophilus*, *Alkalihalobacillus pseudalcaliphilus*, *B. thuringiensis*, *B. anthracis*, *B. pumilus*, *Evansella clarkii*, *Bacillus safensis*, and *Lysinibacillus fusiformis*.

**Figure 5:**
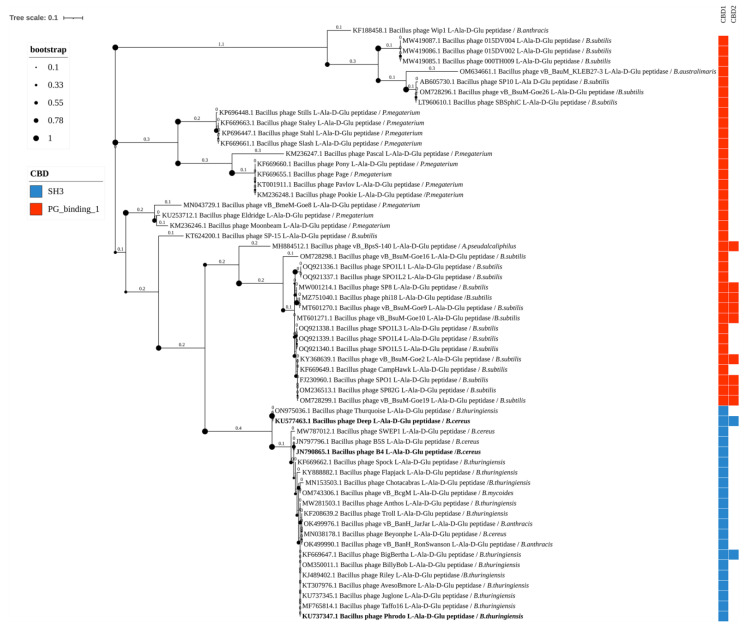
Phylogenetic tree of Peptidase_M15_3 domains from the endolysins of *Bacillus*-infecting bacteriophages. The tree was constructed using the maximum likelihood method (bootstrap analysis with 500 replicates) and visualized in iTOL v.6.9.1. Enzymes previously characterized in scientific publications are highlighted in bold. The host bacteria of the bacteriophages, in whose genomes endolysins were identified, are indicated in italics following the “/” symbol.

Fifty-eight enzymes with the EAD Peptidase_M15_3 domain were identified, which contain two types of CBDs: CH3 and PG_binding_1, forming four different domain combinations (Figure 5). Some members of this group contain two CBDs in one amino acid chain. Only three of these enzymes have been experimentally characterized. The host bacteria of phages with Peptidase_M15_3 domain-containing endolysins include: *B. anthracis*, *B. subtilis*, *B. australimaris*, *P. megaterium*, *A. pseudalcaliphilus*, *B. thuringiensis*, *B. cereus* s.s., and *B. mycoides*.

**Figure 6:**
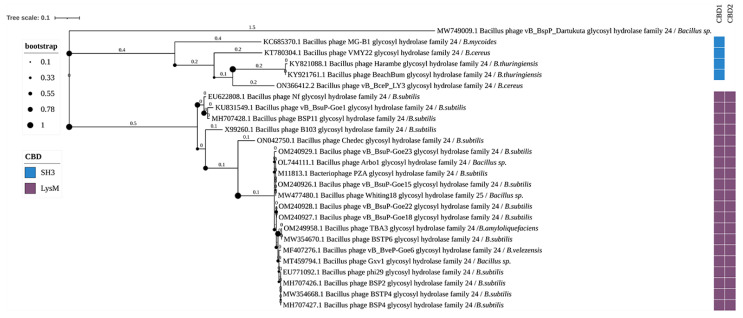
Phylogenetic tree of Bacteriophage_GH24 domains from the endolysins of *Bacillus*-infecting bacteriophages. The tree was constructed using the maximum likelihood method (bootstrap analysis with 500 replicates) and visualized in iTOL v.6.9.1. The host bacteria of the bacteriophages, in whose genomes endolysins were identified, are indicated in italics following the “/” symbol.

Twenty-six enzymes with glycoside hydrolase activity have been identified, possessing the Bacteriophage_GH24 EAD domain and two types of CBDs in two different combinations. These enzymes contain up to two CBDs. To date, none of them have been experimentally characterized (Figure 6). The host bacteria of phages with Bacteriophage_GH24 domain-containing endolysins include: *Bacillus* sp., *B. mycoides*, *B. cereus* s.s., *B. thuringiensis*, *B. subtilis*, *Bacillus amyloliquefaciens*, and *B. velezensis*.

**Figure 7:**
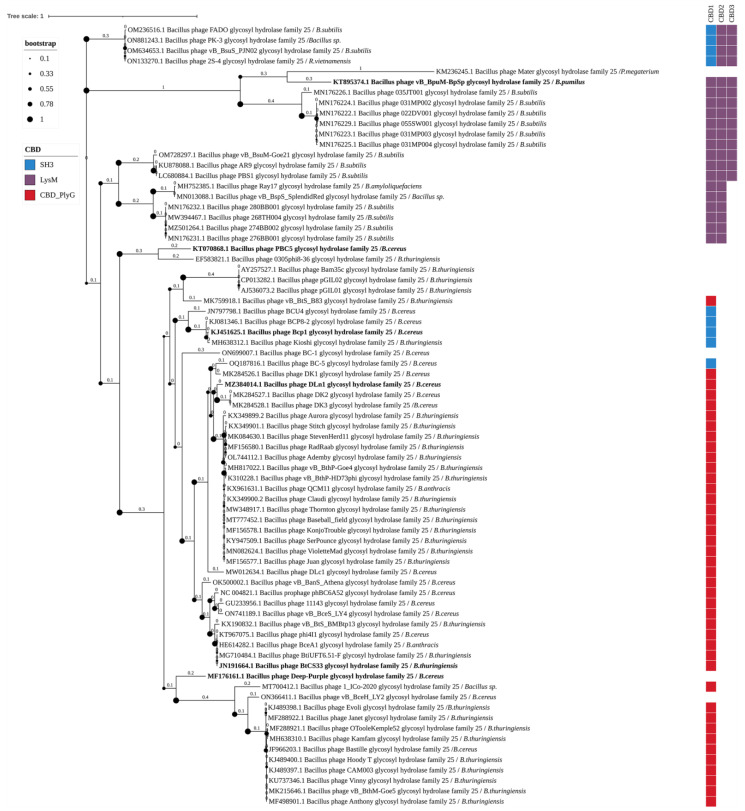
Phylogenetic tree of Glyco_hydro_25 domains from the endolysins of *Bacillus*-infecting bacteriophages. The tree was constructed using the maximum likelihood method (bootstrap analysis with 500 replicates) and visualized in iTOL v.6.9.1. Enzymes previously characterized in scientific publications are highlighted in bold. The host bacteria of the bacteriophages, in whose genomes endolysins were identified, are indicated in italics following the “/” symbol.

Of the 454 enzymes detected, 75 are glycoside hydrolases possessing the EAD Glyco_hydro_25 domain. Six of them have been described in scientific publications (Figure 7). These enzymes contain up to 3 CBDs in one polypeptide chain. Three different types of CBDs were detected in these enzymes: SH3, LysM, and CBD_PlyG, forming six different domain combinations. The bacterial hosts of phages with Glyco_hydro_25 domain-containing endolysins include: *B. subtilis*, *Bacillus* sp., *Rossellomorea vietnamensis*, *P. megaterium*, *B. pumilus*, *B. amyloliquefaciens*, *B. cereus* s.s., *B. thuringiensis*, and *B. anthracis*.

**Figure 8:**
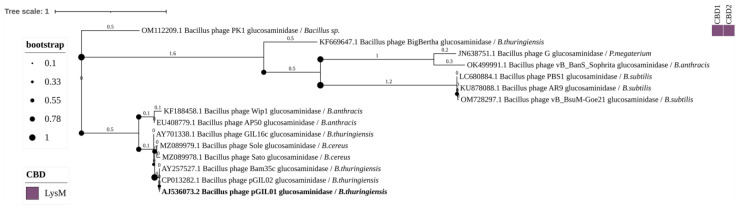
Phylogenetic tree of Glycosyl_Hydrolase_73 domains from the endolysins of *Bacillus*-infecting bacteriophages. The tree was constructed using the maximum likelihood method (bootstrap analysis with 500 replicates) and visualized in iTOL v.6.9.1. Enzymes previously characterized in scientific publications are highlighted in bold. The host bacteria of the bacteriophages, in whose genomes endolysins were identified, are indicated in italics following the “/” symbol.

We also identified 15 N-acetylglucosaminidases with the EAD Glycosyl_Hydrolase_73, however, CBDs were not detected in 14 of them. The bacteriophage PK1 endolysin was found to have two CBDs, which belong to the LysM domain type (Figure 8). In this group of endolysins, only the bacteriophage pGIL01 endolysin was experimentally characterized. Bacterial hosts of phages with Glycosyl_Hydrolase_73 endolysins include: *Bacillus* sp., *B. thuringiensis*, *P. megaterium*, *B. anthracis*, *B. subtilis*, and *B. cereus* s.s.

## 4. Discussion

In three of the 438 genomes examined, genes encoding endolysins were not detected: *Bacillus* phage GaW-2019a (CP042877.1), *Bacillus* phage Hybphi3Ts (CP052843.1), and *Bacillus* phage vB_Bacillus_1020A (MT210152.1). One reason for this may be that the endolysins of these bacteriophages have unique sequences and their homologs are absent from the databases used for annotation. It may also be the case that only a partial assembly of the genomes of these phages was available for analysis, as a result of which some genes were missing. The genome sequence of phage GaW-2019a is only 7379 bp long, and that of phage Hybphi3Ts is 31,849 bp long. Despite the fact that the first genome was presented in the database as ‘complete’ and the second as ‘partial’, in both cases, endolysin genes were not detected.

### 4.1. N-Acetylmuramoyl-L-Alanine Amidase

N-acetylmuramoyl-L-alanine amidase (NALAA) or amidases for short, catalyze the cleavage of the bond between N-acetylmuramic acid and L-alanine residues. They represent one of the most common families of bacterial amidases and are divided into three types: N-acetylmuramoyl-L-alanine amidase type 2, N-acetylmuramoyl-L-alanine amidase type 3, and N-acetylmuramoyl-L-alanine amidase type 5. Each of these types has a corresponding EAD: Amidase_2, Amidase_3, and Amidase_5 [24]. Among the endolysins identified in this work, only members of the Amidase_2 and Amidase_3 types were found.

#### 4.1.1. Amidase_2 Domain-Containing Endolysins of *Bacillus*-Infecting Bacteriophages

Type 2 N-acetylmuramoyl-L-alanine amidases are characterized by the presence of binding sites for divalent Zn^2+^ ions (PDB 2L47). Among the endolysins found in this work, 193 EADs belong to the Amidase_2 type, which makes this group the most numerous. According to the obtained data, endolysins of *Bacillus*-infecting bacteriophages of the Amidase_2 type can contain up to three CBDs, which can belong to six different types: SH3 domain (found in 90 enzymes), LysM domain (in 7), CBD_PlyG domain (in 17), PG_binding_1 domain (in 11), DUF3597 domain (in 35), and SPOR domain (in 1). At the same time, CBDs were not detected in 36 endolysins of this group, which may be due to such CBD domains not having been identified yet and/or are missing in the databases that were searched. Among all of the identified groups of endolysins, those containing the Amidase_2 EAD demonstrated the greatest diversity in both CBD types and the bacterial hosts of phages.

In the phylogenetic tree constructed based on the amino acid sequences of the detected Amidase_2 domains, the clades correlated with both the CBD types of the enzymes (although their CBD sequences were not used for phylogenetic inference) and with their bacterial hosts (Figure 3). Although bacteriophages can often infect several bacterial species, their specificity range rarely extends beyond one genus. The phylogenetic tree clearly shows (Figure 3) the Amidase_2 domains from the endolysins of phages targeting the *Bacillus cereus sensu lato* (s.l.) group into separate clades. These amidases 2 contain SH3 and CBD_PlyG domains as CBD. A related branch is formed by Amidase_2 domains of the endolysins of *Paenibacillus* bacteriophages, in which CBDs have not been identified. Amidase_2 domains from the endolysins of phages infecting *B. subtilis* form several branches, both close to *Bacillus cereus* s.l. and distant, and contain the SH3, LysM, PG_binding_1. and DUF3597 types of CBDs. The DUF3597 domain was found only in the endolysins of phages infecting *B. subtilis* and *B. pumilus*. Amidase_2 domains from the endolysins of phages infecting other bacterial species are rare, and in the phylogenetic tree, they either group together with Amidase_2 domains of phage endolysins against *Bacillus cereus* s.l. and *B. subtilis,* or form distinct clades. Of all the Amidase_2 domain-containing endolysins, only 13, from phages infecting *Bacillus cereus* s.l., have been characterized, while those from phages of other bacterial species remain uncharacterized.

The endolysin PlyG of phage gamma was first shown to be an effective antibacterial agent for systemic treatments in a mouse model of bacteremia caused by *B. anthracis* [25]. PlyG is a two-domain endolysin consisting of the Amidase_2 (PDB 2L47) and CBD_PlyG (PDB 2L48) domains. This enzyme is active against the vegetative cells and germinating spores of *B. anthracis* and *B. cereus* s.s. [26]. In addition to the two main domains, PlyG has a spore-binding domain, which is part of the EAD sequence and ensures the binding of PlyG to the exosporium of *B. anthracis*. Despite the presence of a spore-binding domain, PlyG has not been shown to be capable of destroying the spore form of *B. anthracis* [27]. It has also been established that the presence of CBD is necessary for the bacteriolytic activity of PlyG [28].

Two endolysins, pLYS250 from the Tp250 prophage of *B. cereus* 250 and LysEFR-4 from the PfEFR-4 prophage of *B. cereus* EFR-4, are 95% identical to each other and have a domain composition similar to that of PlyG. The sequence identity of pLYS250 and LysEFR-4 endolysins to PlyG is 46% and 47%, respectively. There are no data on the properties of pLYS250 in the scientific literature [29]. LysEFR-4 demonstrates the ability to lyse emetic isolates of *B. cereus* s.s. and *B. mycoides* as well as some strains of *B. thuringiensis*, *B. subtilis*, *Lysinibacillus sphaericus*, and *Salmonella enterica* [30].

Four endolysins of phages infecting *B. cereus* s.s. contain two CBDs of the SH3 type: PlyHSE3 of bacteriophage vB_BceM-HSE3, LysPW2 of bacteriophage PW2, LysPBC2 of bacteriophage PBC2, and Ply57 of bacteriophage Izhevsk. All of these endolysins are active against strains of *B. cereus* s.s., *B. anthracis*, and *B. thuringiensis*, but differ in specificity when tested against other bacterial species. PlyHSE3 was active against *Pseudomonas aeruginosa* (in the presence of EDTA); LysPW2 against strains of *B. mycoides*, *B. paranthracis*, and *B. subtilis*; LysPBC2 against *B. mycoides*, *B. subtilis*, *P. megaterium*, *Niallia circulans*, *B. licheniformis*, *B. pumilus*, *Listeria monocytogenes*, and *Clostridium perfringens*; and Ply57 against *B. mycoides*, *B. flexus*, *B. subtilis*, and *P. megaterium* [31]. Similarly to PlyG, the EAD of LysPW2 did not demonstrate lytic activity in the absence of CBDs, but was able to bind to the spores of *B. cereus* s.s. [32]. The EAD of LysPBC2 alone was capable of lysing a wide range of bacteria, however, its activity against *B. cereus* s.s. was lowered compared to the full-length enzyme. The EAD of LysPBC2 also showed the ability to bind to *B. cereus* spores, but not to other species [33]. At 55 °C, the full-length Ply57 endolysin was two times more stable than the endolysin PlyG [34].

Endolysins LysBPS13 of bacteriophage BPS13 (*B. cereus* s.s.) and PlyN74 of bacteriophage Nigalana (*B. thuringiensis*) contain one CBD of the SH3 domain type. LysBPS13 demonstrated bacteriolytic activity against strains of *B. cereus* s.s., *B. thuringiensis*, *B. licheniformis*, *P. megaterium*, and *B. pumilus*, and in the presence of EDTA, against *Salmonella*, *Escherichia coli*, *Cronobacter sakazakii*, and *Shigella*. The presence of EDTA did not affect the enzyme activity, despite the presence of Zn^2+^-binding residues in this enzyme. LysBPS13 is characterized by high thermal stability, especially in the presence of 30% glycerol: heating at 100 °C for 30 min reduced its bacteriolytic activity, but not more than by 85% compared to the unheated control sample. Autoclaving at 121 °C for 15 min completely inactivated the enzyme [35]. Endolysin PlyN74 demonstrated bacteriolytic activity against *B. cereus* s.s., *B. thuringiensis*, *B. amyloliquefaciens*, *N. circulans*, *B. licheniformis*, *P. megaterium*, *B. pumilus*, *B. anthracis*, and *L. sphaericus*. Heating at 60 °C reduced the activity of PlyN74 to less than 10% of the initial level. At the same time, overnight treatment with EDTA did not affect the activity of PlyN74 [36].

The endolysin LysIS075 of phage PfIS075 of *B. cereus* s.s. contains CBD_PlyG. LysIS075 demonstrated bacteriolytic activity against *B. cereus* s.s., *B. thuringiensis*, *B. anthracis*, and *B. mycoides*. LysIS075 was able to reduce the number of colony-forming units (CFUs) of emetic *B. cereus* s.s. strains in rice porridge and milk by 80% and 60%, respectively [37].

Endolysins LysJ and LysF of *B. anthracis*-infecting bacteriophages J5a and F16Ba, respectively, contain an SH3 domain and a signal peptide (SP) at the N-terminus. The presence of SP allows such enzymes to destroy the cell from the inside without the need for a holin. Both endolysins were active against some strains of *B. anthracis*, *B. cereus* s.s., *B. thuringiensis*, *B. mycoides*, and *B. subtilis*. However, the activity differed depending on the method: when analyzed by spot test, the enzymes acted only on *B. anthracis*, whereas when analyzed by the turbidimetric method, they were also active against all of the above-mentioned species. The lytic spectrum of LysJ matched that of its source phage, whereas the lytic spectrum of LysF was narrower compared to its source phage, and the endolysin was used in a higher concentration to achieve a visible effect [38].

The endolysin PlyBMB of bacteriophage vB_BthS_BMBphi infecting *B. thuringiensis* contains an uncharacterized CBD. The enzyme was active against *B. cereus* s.s., *B. thuringiensis* and *B. anthracis*, and also exhibited low lytic activity against *B. pumilus* and *B. subtilis* strains [39].

Thus, the experimentally characterized Amidase_2 domain-containing endolysins of *Bacillus*-infecting bacteriophages demonstrated different ranges of bacteriolytic activity, with most of them being active against *Bacillus cereus* s.l. Some of the endolysins were active against members of other bacterial genera from the *Bacillaceae* family as well as other families. Two enzymes, LysBPS13 and PlyN74, were shown to retain their bacteriolytic activity in the presence of EDTA. The Amidase_2 domains, without CBDs, either completely lost their activity or retained it, sometimes with a change in the specificity range compared to the full-length enzyme. The Amidase_2 domains of three endolysins, PlyG, LysPW2, and LysPBC2, were capable of binding to the spores.

#### 4.1.2. Amidase_3 Domain-Containing Endolysins of *Bacillus*-Infecting Bacteriophages

Type 3 N-acetylmuramoyl-L-alanine amidases, similar to type 2 amidases, are zinc-dependent enzymes (7B3N). Of the 83 type 3 amidases identified in this work, only five have been experimentally characterized (Figure 4). Similarly to the type 2 amidases, type 3 amidases can contain 6 different types of CBDs: LysM (found in 32 endolysins), SH3 (in 19), CBD_PlyG (in 11), DUF5776 (in 6), SPOR- (in 4), and PG_binding_1 (in 2). In nine endolysins, no CBD was detected. These enzymes have been identified in the genomes of phages that infect a wide variety of bacterial hosts.

In the phylogenetic tree of the identified Amidase_3 domains, the domains can be clearly divided into three large clusters with subclusters (Figure 4). Some correlation with the CBD types and the bacterial hosts of phages was observed in these clusters (Figure 4). The first cluster was an exception: it contained five sequences with LysM domains, two sequences with no identified CBDs, and one sequence with an SH3 domain. The endolysins containing Amidase_3 domains from cluster 1 belonged to phages that act against different bacterial species: *B. cereus* s.s., *Bacillus* sp., *P. megaterium*, *B. subtilis*, and *A. pseudalcaliphilus*. Within this cluster, the Amidase_3 were not closely related, which is not surprising since the endolysins containing them are found in unrelated phages. The second cluster was also diverse but was significantly larger than the first one. It included mainly Amidase_3 domains of endolysins with the LysM and DUF5776 domains from *B. subtilis* and *B. pumilus*-infecting phages and also included Amidase_3 domains of endolysins from phages of other bacteria including *B. cereus* s.s. and *B. thuringiensis*. The third cluster consisted exclusively of Amidase_3 domains of endolysins from phages infecting *B. cereus* s.l. In contrast to type 2 amidases characteristic of *Bacillus* phages, type 3 amidases can contain not only SH3 and PlyG, but also SPOR, DUF5776, and LysM types of CBDs. All five experimentally characterized Amidase_3 domain-containing endolysins of *Bacillus*-infecting phages were from the third cluster.

One of the experimentally characterized Amidase_3 domain-containing endolysins is AP50-31, from phage AP50 infecting *B. anthracis*. In 2018, it was described as containing Amidase02_C (CBD) and Amidase_3 (EAD); however, structural analysis with InterPro revealed a CBD_PlyG domain rather than Amidase02_C (a previously used domain name) as the CBD. In some other publications, the former designation of the CBD_PlyG domain is used. AP50-31 exhibited bacteriolytic activity against *B. cereus* s.s., *B. licheniformis*, *B. subtilis*, *B. thuringiensis*, and *B. anthracis* [40].

Endolysin LysPBC1, consisting of the same domains as AP50-31, belongs to bacteriophage PBC1 of *B. cereus* s.s. LysPBC was active against *B. cereus* s.s., *B. thuringiensis*, *B. mycoides*, *P. megaterium*, *B. subtilis*, *B. licheniformis*, *L. sphaericus*, *N. circulans*, and in the presence of EDTA, against *E. coli*, *Shigella flexneri*, and *C. sakazakii*. The EAD of LysPBC, isolated separately, had a narrower specificity range and lower activity compared to the full-length enzyme when tested against *B. cereus* s.l. At the same time, against *B. subtilis* strains, it showed higher activity compared to the full-length enzyme [41].

Endolysin LysPBC4 belongs to bacteriophage PBC4 of *B. cereus* s.s., and consists of an Amidase_3 EAD and a CBD_PlyG. The enzyme was active against *B. cereus* s.s., *B. thuringiensis*, and *B. mycoides* [42].

LysPW4 from bacteriophage pW4 of *B. cereus* s.s. contains a CBD_PlyG CBD, which is 79.5% identical to that in LysPBC4. LysPW4 was active against *B. cereus* s.s., *B. paranthracis*, and *B. mycoides*. The lytic activity range of the enzyme was narrower than that of its phage. In the absence of the CBD, the catalytic domain of LysPW4 did not exhibit bacteriolytic activity, however, it remained capable of binding to spores, though not to vegetative cells [32].

PlyTB40 of the *B. cereus* s.s. phage TsarBomba contains an SH3 type CBD. The enzyme was similar in activity to PlyN74, a type 2 amidase. PlyTB40 was completely inhibited by heating at 60 °C for 30 min, while treatment with EDTA had no impact on its activity. PlyTB40 demonstrated bacteriolytic activity against *B. cereus* s.s., *B. thuringiensis*, *B. amyloliquefaciens*, *B. circulans*, *B. licheniformis*, *P. megaterium*, *B. pumilus*, *B. anthracis*, and *L. sphaericus* [36].

Thus, type 2 and type 3 N-acetylmuramoyl-L-alanine amidases of bacteriophages infecting *B. cereus* s.l. can bind to spores and retain their activity when treated with EDTA, which may be important characteristics in the development of endolysin-based drugs. Methods for applying such endolysins in the food industry and medicine are being tested. Amidases of phages infecting other bacilli have not yet been characterized experimentally. These enzymes contain various combinations of domains, and their biological potential has to be thoroughly studied. More in-depth studies can reveal new enzyme properties and hence new possibilities for their use.

### 4.2. Peptidases of Bacillus-Infecting Bacteriophages

Peptidases include endolysins capable of cleaving peptide bonds in the peptide chains of peptidoglycan. Endolysins of *Bacillus*-infecting phages possess different EADs with a protease activity, in particular, Peptidase_M15_3 and NLPC_P60.

#### 4.2.1. Peptidases M15

Peptidases M15 are a family of zinc-containing carboxypeptidases that includes four subfamilies that differ in the type of bond they hydrolyze. These enzymes are mainly found in bacteria and protozoa [43]. In this work, 58 enzymes were found that contained Peptidase_M15_3 domains and up to 2 CBDs that can belong to 2 different types: 36 endolysins had PG_binding_1 and 31 had SH3. One endolysin contained no identifiable CBD (Figure 5). The identified Peptidase_M15_3 domain-containing endolysins belonged to phages infecting eight bacterial species: *B. anthracis*, *B. subtilis*, *B. australimaris*, *P. megaterium*, *A. pseudalcaliphilus*, *B. thuringiensis*, *B. cereus* s.s., and *B. mycoides*. This group contained 12 endolysins of phages infecting *P. megaterium*, while in the other groups, there were only 1–2 such endolysins.

In the phylogenetic tree of the amino acid sequences of the identified Peptidase_M15_3 domains, three clusters could be distinguished (Figure 5). The first cluster included the EAD of endolysins from phages infecting *B. subtilis*, *B. australimaris*, and *B. anthracis*. No CBD was found in the endolysin of phage Wip1 infecting *B. anthracis*, and the remaining endolysins had a PG_binding_1 CBD. The second cluster included Peptidase_M15_3 domains of endolysins from phages infecting *P. megaterium*, with these endolysins containing PG_binding_1 CBDs. The third Peptidase_M15_3 cluster could be divided into three subclusters. The first subcluster contained three EAD sequences obtained from phages of *P. megaterium*, whose endolysins featured PG_binding_1 CBDs. The second subcluster was composed of EAD sequences obtained from the endolysins of *B. subtilis* phages also with PG_binding_1 domains. The third subcluster included Peptidase_M15_3 domains of endolysins from phages infecting *B. cereus* s.l., which contained SH3-type domains as their CBDs. The only three experimentally characterized M15 peptidases of *Bacillus*-infecting phages belonged to the third subcluster.

LysB4 of bacteriophage B4 is the first experimentally characterized endolysin of a *Bacillus*-infecting phage that has a Peptidase_M15_3 domain. The enzyme exhibits its maximum activity under alkaline conditions, in the pH range of 8.0–10.0. LysB4 is deactivated by heating at 55 °C for 30 min. It was also shown that the enzyme lost its activity when treated with EDTA, but that its activity was restored when Zn^2+^, Mn^2+^, Ca^2+^ and Mg^2+^ ions were added. LysB4 effectively lysed strains of *B. cereus* s.s., *B. subtilis*, and *L. monocytogenes*, and was capable of lysing other species pretreated with EDTA such as *E. coli*, *P. aeruginosa*, *C. sakazakii*, *S. enterica*, *S. flexneri*, and *Shigella boydii* [44].

The endolysin PlyP56 of the bacillary bacteriophage Phrodo has the same domain composition as LysB4: peptidase_M15_3 EAD and SH3-type CBD. PlyP56 was experimentally compared to PlyN74 and PlyTB40, which also had SH3-type CBDs, but possessed different EADs—Amidase_2 and Amidase_3, respectively. The SH3 domains of PlyN74 and PlyTB40 were 94% and 50% identical to that of PlyP56. The range of sensitive *Bacillus* strains was identical for PlyP56, PlyN74, and PlyTB40, which is probably due to the presence of homologous CBDs. However, PlyP56 demonstrated significantly higher activity against *B. tropicus* ATCC 4342 compared to PlyN74 and PlyTB40. Compared to PlyN74 and PlyTB40, the activity of PlyP56 decreased more when heated to 55 °C. Similarly to LysB4, PlyP56 was inactivated by EDTA treatment, but the activity was restored by the addition of Mg^2+^ and Ca^2+^ ions [36].

The endolysin PlyB221 of phage Deep-Blue infecting *B. cereus* s.s. has a Peptidase_M15_3 EAD and two SH3 type CBDs. The enzyme was active against bacteria of the *B. cereus* s.l. as well as *B. licheniformis*, *B. pumilus*, and *P. megaterium*. PlyB221 retains its lytic activity when heated to 50 °C [45].

#### 4.2.2. NlpC/P60 Peptidases

Another group of phage endolysins identified in this work belong to the NlpC/P60 hydrolase superfamily (named after NlpC, a new lipoprotein C from *E. coli*, and P60, a 60-kDa protein from *Listeria monocytogenes*). The presence of the NlpC_P60 domain is characteristic of these enzymes. These hydrolases are D,L-endopeptidases and hydrolyze D-γ-glutamyl-meso-diaminopimelate bonds in the cell wall peptides [46]. The crystal structure of one such enzyme, the NlpC_P60-containing peptidase from *B. cereus* s.s. with two SH3 domains, has been solved (PDB 3H41). It was found that such peptidases can possess a combination of the NlpC_P60 domain with other EADs such as amidase. They also have different types and varying quantities of CBDs. In this work, only three NlpC_P60 domain-containing peptidases were identified, therefore no phylogenetic tree was built for this group. In the enzyme sequences, CBDs were not detected. The identified peptidases included those of phage Palmer (KP411017.1) infecting *P. megaterium*, phage Maceta (MH538296.1) infecting *B. thuringiensis*, and phage MG-B1 (KC685370.1) infecting *B. mycoides*. Notably, phage MG-B1 also encoded an endolysin from the glycoside hydrolase family 24, with the Bacteriophage_GH24 type of EAD.

Thus, the endolysins of *Bacillus*-infecting bacteriophages that belong to the peptidase class can be divided into two groups: peptidases M15 and NlpC/P60. Three M15 peptidases have been characterized, and it was shown that they require Zn^2+^ ions for bacteriolytic activity, and lose the activity upon treatment with EDTA or heating above 55 °C. Peptidases of phages infecting *B. cereus* s.l. are only a fraction of such enzymes that can be found in all *Bacillus*-infecting phages. Most endolysins of *P. megaterium* phages are peptidases. The NlpC/P60 group includes only three enzymes that remain experimentally uncharacterized, and whose CBDs were not identified bioinformatically in the course of this work.

### 4.3. Glycoside Hydrolases

Glycoside hydrolases are enzymes that break down glycosidic bonds in polysaccharides. There are more than 150 families of glycoside hydrolases [47]. Bacteriophages were found to contain N-acetylglucosaminidases from families 3 and 73 as well as N-acetylmuramidases belonging to families 22, 24, and 25 [24]. In this study, we found EADs within the endolysins of *Bacillus* phages that were typical of three glycoside hydrolases families: family 73, containing the Glycosyl_Hydrolase_73 domain, and families 24 and 25 with the Bacteriophage_GH24 and Glyco_hydro_25 domains, respectively.

#### 4.3.1. Glycoside Hydrolase Family 24 (GHF24)

GHF24 contains Bacteriophage_GH24 EADs. The crystal structure of one endolysin from this family, the AbLys1 hydrolase, has been solved. The EAD of AbLys1 consists of six α-helices and a β-hairpin (PDB 8APP). This enzyme has been shown to be a promising agent for combating *Acinetobacter baumannii* [48]. In this work, we found 26 *Bacillus*-phage enzymes from this family, none of which have been experimentally characterized to date. In the phylogenetic tree of the selected Bacteriophage_GH24 domain, there were two clusters and a separate singleton—the domain sequence obtained from the genome of bacteriophage vB_BspP_Dartukuta (Figure 6). No CBD was identified in the vB_BspP_Dartukuta endolysin.

The first cluster in the tree contained five domains, all from five endolysins of phages infecting *B. cereus* s.l. Four of the Bacteriophage_GH24-containing endolysins contained SH3-type CBDs, and one endolysin had no identifiable CBD. Domains in the second cluster were all from endolysins containing two LysM CBDs. Most endolysins with EADs from the second cluster belonged to phages infecting *B. subtilis*, and only a few to phages infecting *Bacillus* sp., *B. amyloliquefaciens*, and *B. velezensis*. Thus, glycoside hydrolase family 24 are more characteristic of phages infecting *B. subtilis* than other *Bacillus* species.

#### 4.3.2. Glycoside Hydrolase Family 25 (GHF25)

GHF25 are enzymes with lysozyme activity that contain the Glyco_hydro_25 domain type of EAD [24]. The three-dimensional structure of GHF25 features the (β/α)_5_(β)_3_ TIM-like domain (PDB 2NW0) [47]. Of the 454 selected endolysins, 75 belonged to GHF25. Their EADs were divided into three clusters with subclusters (Figure 7). The first cluster included four EADs from endolysins, containing one SH3 domain and two LysM domains as CBDs. These endolysins belonged to phages infecting *B. subtilis*, *Bacillus* sp., and *R. vietnamensis*. The second cluster contained eight EADs from endolysins, seven of which each had three LysM domains, and one had no identifiable CBD. One of the endolysins with the Glyco_hydro_25 domain of the second cluster has been experimentally characterized (Figure 7). The third cluster was the largest and included the remaining 63 sequences, of which 54 belonged to phages infecting *B. cereus* s.l. These endolysins contained either an SH3 domain (5 enzymes) or a CBD_PlyG (41 enzymes), and in the other 7 of these endolysins, no CBDs were detected. Notably, the nine endolysins of phages infecting other *Bacillus* species contained LysM domains. Five endolysins, whose EADs belonged to the third cluster, have been experimentally characterized to date. GHF25 are more commonly present in phages infecting *B. cereus* s.l. than GHF24, and were the second most frequently mentioned type of endolysin of the *Bacillus*-infecting phages.

Endolysin LysDLn1 of phage DLn1 infecting *B. cereus* s.s. is a GHF25, which has a CBD_PlyG domain. The specificity range of this enzyme is much broader than that of its bacteriophage: the phage lysed 16 of 122 *B. cereus* s.s. strains, while the endolysin lysed 82 of them. LysDLn1 also reduced the CFUs of *B. cereus* s.s. in milk, and the effect remained after 24 h, which was not the case with the phage. Thus, the endolysin may be a more promising agent for food preservation compared to the bacteriophage [49]. Endolysins similar to LysDLn1 were found in bacteriophages DK1, DK2, and DK3, but have not yet been characterized [50].

The endolysin PlyBt33 of bacteriophage BtCS33 infecting *B. thuringiensis* has a similar structure to LysDLn1: Glyco_hydro_25 EAD and CBD_PlyG CBD. PlyBt33 lysed *B. thuringiensis*, *B. subtilis*, *B. pumilus*, *B. cereus* s.s., and *B. anthracis* strains. The EAD of PlyBt33 alone exhibited bacteriolytic activity, though significantly weaker compared to the full-length enzyme. The CBD of the enzyme was shown to bind to *B. thuringiensis* and *B. subtilis* cells [51].

The endolysin LysPBC5 of phage PBC5 infecting *B. cereus* s.s. has an unclassified type of CBD, probably due to the lack of described homologs. However, its CBD has shown the ability to bind to *B. cereus* s.s. and *B. thuringiensis* cells [52].

PlyP32 of phage Deep-Purple infecting *B. cereus* s.s. contains an SH3 type CBD. This was active against bacteria of the *B. cereus* group as well as strains of *B. licheniformis*, *B. pumilus*, and *P. megaterium*. The specificity range of PlyP32 was wider than that of its phage. The CBD of PlyP32 was able to bind only to *B. cereus* s.l. The enzyme has a narrow pH stability range: at pH 7.0 the activity is 100%, at pH 8.0 it decreases to 30%, at pH 6.0 it is 80%, and at pH 5.0 it is 30%. NaCl at a concentration above 300 mM and a temperature of 60 °C completely deactivate the enzyme [45].

PlyB of bacteriophage vB_BanS_Bcp1 infecting *B. anthracis* contains an SH3 type CBD. The endolysin was active against *B. cereus* s.l., including *B. anthracis* strains. PlyB demonstrated a lower rate of resistance development compared to conventional antibiotics: serial passages of *B. tropicus* ATCC 4342 for 20 days with increasing concentrations of ciprofloxacin and PlyB led to a 64-fold increase in the minimum inhibitory concentration of ciprofloxacin, and only a 2-fold increase in the case of PlyB. Interestingly, a dose-dependent synergistic effect was described for PlyB and PlyG: co-administration of these endolysins both in vivo and in vitro enhanced their activity, probably due to their different EADs and CBDs [53].

Gp255 of phage vB_BpuM_BpSp infecting *B. pumilus* contains three LysM type CBDs. The enzyme demonstrated high bacteriolytic activity against *B. pumilus*, weak against *B. subtilis* and *B. anthracis*, and no activity against other bacteria including *B. cereus* s.s. The enzyme was thermally stable, retaining approximately 15% of its activity after heating at 100 °C for 10 min [54].

#### 4.3.3. Glycoside Hydrolase Family 73 (GHF73)

GHF73 are N-acetylglucosaminidases, also known as mannosyl-glycoprotein endo-β-N-acetylglucosaminidases [55]. In this work, 15 enzymes belonging to this family were found, in 14 of which the CBDs were not identified (Figure 8). The endolysin of phage PK1 contains two CBDs of the LysM type and was the only endolysin of this group for which no genes of other endolysins were found in the genome of its bacteriophage. Most endolysins of the GHF73 group belonged to phages infecting *B. cereus* s.l. According to a zymogram method, the endolysin of phage pGIL01 infecting *B. thuringiensis* demonstrated hydrolase activity against peptidoglycan of next bacteria: *B. thuringiensis*, *B. subtilis*, and *Micrococcus luteus* [56].

Thus, of the three described glycoside hydrolases of *Bacillus* phages, only GHF25 contains experimentally characterized endolysins. Some GHF25 have been shown to reduce the CFUs of *B. cereus* s.s. in milk, and to treat *B. anthracis* infection in mice. The endolysins of the GHF24 and GHF73 are poorly studied, and their potential as antibacterial agents is still unknown.

### 4.4. MLTF Proteins

The last group of enzymes identified in this study were the MLTF-like proteins (lytic murein transglycosylase F). MLTF-like proteins belong to family 1 of lytic transglycosylases. They are lytic transglycosylases, which cleave the glycosidic bond between N-acetylmuramyl and N-acetylglucosamine to form 1,6-anhydromuramyl [57]. In this work, only one such enzyme was found, encoded by bacteriophage vB_BspM_AgentSmith infecting *Bacillus* sp. Unlike other endolysins of *Bacillus*-infecting bacteriophages, MLTF proteins are not hydrolases.

### 4.5. CBDs of Endolysins of Bacillus-Infecting Phages

Currently, CBDs are divided into four groups: CW_binding_1 (choline-binding), SH3 (SH3b), triple-helix bundles (CW_7 and PG_binding_1), and α/β structural [58].

In 76 of the 454 endolysins analyzed in this work, no CBDs were detected. This may be due to both the limitations of bioinformatic methods, and the fact that such domains have not yet been identified in the enzymes at all. In some endolysins, CBDs were not determined in our analysis, although they were previously mentioned in the corresponding publications on the particular endolysins. In total, we found seven types of CBDs in the *Bacillus*-infecting phages: SH3, which was the most common, LysM, CBD_PlyG, PG_binding_1, DUF3597, DUF5776, and SPOR.

#### 4.5.1. The SH3 Domain

SH3 domains were first described in 1988 in eukaryotic proteins involved in intercellular communication as well as intracellular signaling from the cell surface to the nucleus [59]. This domain is one of the best-studied CBDs, and is present in different proteins from a wide variety of living organisms. SH3 domains typically form a β-barrel structure consisting of 5–7 β-strands, which are often arranged as two antiparallel β-sheets connected by linkers [58]. Typically, this domain binds to proline-rich binding regions. The affinity of SH3 domains to their peptide ligands is quite low, and so is their selectivity, however, these domains play important roles in complex regulatory interactions [60].

SH3 domains were found in 143 of the selected 454 endolysins including N-acetylmuramoyl-L-alanine amidases, peptidases M15, GHF24, and GHF25. In these enzymes, SH3 domains were present either as single CBDs, in pairs, or in a combination with other types of CBDs. Sixteen of the 143 SH3 domain-containing endolysins have been described in scientific publications.

The SH3 domains of endolysins of bacteriophages vB_BanS-Tsamsa and W.Ph. were considered for use in test systems designed to detect bacilli in food products. These domains demonstrated a similar binding range on *Bacillus* strains. They were linked to green fluorescent protein (GFP), biotinylated at the N-terminus and applied to Dynabeads^®^ magnetic particles coated with streptavidin. When such systems were tested on food samples, the sensitivity of the magnetic particles with a single SH3 domain was low: at concentrations below 10^2^ cells/gram or ml, cells were not detected. However, when magnetic particles with an SH3 domain were combined with particles with other types of CBDs, the sensitivity significantly increased. Magnetic particles with different CBDs combined showed the best sensitivity in milk and reconstituted infant formula, and more than 10 times lower sensitivity in spices, milk rice, or wheat flour [61].

#### 4.5.2. The LysM Domain

The LysM domain is found in both prokaryotes and eukaryotes and consists of several α-helices and β-sheets. This domain was first discovered in *Bacillus* bacteriophage φ29 and then repeatedly described in peptidoglycan hydrolases of both bacteria and phages. These domains are often found in proteins in the form of several repeating CBDs connected by short linker regions [62]. In this work, LysM domains were detected in 80 endolysins, of which only one has been described in the scientific literature: Gp255 of bacteriophage vB_BpuM_BpSp. Gp255 contains the Glyco_hydro_25 EAD and three LysM CBDs [54]. In endolysins of *Bacillus*-infecting phages, LysM domains can present as single CBDs, in pairs or triplets, or in a combination with other types of CBDs. In this study, LysM domains were only detected in the N-acetylmuramoyl-L-alanine amidases and glycoside hydrolases, while no LysM domains were detected in peptidases.

#### 4.5.3. The CBD_PlyG Domain

The CBD_PlyG domain was named after the PlyG endolysin, one of the first endolysins proposed for the treatment of anthrax [25]. This domain belongs to α/β structures and in some publications is referred to as Amidase02_C, the truncated version of Amidase_2 [40]. In this work, 69 endolysins containing the CBD_PlyG domain were identified including 10 experimentally characterized enzymes. In these 69 enzymes, there was only one copy of CBD_PlyG, unlike the previously described SH3 and LysM, which were found in pairs and triplets, and in combinations with other CBDs. The CBD_PlyG domain is capable of binding to vegetative forms of *Bacillus* cells. In endolysins of phages infecting other bacterial genera, this domain has not been detected. The CBD_PlyG domain was found in the autolysins of *B. thuringiensis* subsp. *israelensis* and *B. anthracis* Ames [63,64]. In this work, the CBD_PlyG domain was found in type 2 and type 3 N-acetylmuramoyl-L-alanine amidase, and in glycoside hydrolase family 25.

#### 4.5.4. The PG_Binding_1 Domain

The PG_binding_1 domain consists of three alpha helices. This domain was shown to bind to peptidoglycan. The PG_binding_1 domain has been found at both the N- and C-termini of various enzymes involved in bacterial cell wall degradation. An experimentally characterized endolysin containing this domain is Lytμ1/6 of bacteriophage μ1/6 infecting *Streptomyces aureofaciens*. Peptidoglycan-binding activity of both the full-length and truncated variants of Lytμ1/6 has been demonstrated [65].

In this work, the PG_binding_1 domain was found in 48 of the selected 454 endolysins. These 48 endolysins include N-acetylmuramoyl-L-alanine amidases and peptidases. The PG_binding_1 domain in these enzymes occurs as a single CBD, two or three repeating CBDs, or in combination with other types of CBDs. The only experimentally described PG_binding_1 domain is that of the peptidase M15 of bacteriophage SPO1. The SPO1 domain was used to develop a test system for detecting *Bacillus* in food. The binding efficiency of magnetic particles with the SPO1 PG_binding_1 domain to tested *Bacillus* strains was lower than that of magnetic particles with CBDs from endolysins of Tsamsa and W.Ph. For the purpose of detecting *Bacillus* in food products, magnetic particles with the SPO1 PG_binding_1 domain were insufficient. However, the combined use of magnetic particles with CBDs from endolysins of the SPO1 and Tsamsa phages significantly improved the sensitivity of the test system [61].

The uncharacterized CBD of the bacteriophage SPP1 endolysin, marked in InterPro as the DUF5776 domain with a putative peptide-binding function, was also used in a test system. In its level of binding activity, this domain was comparable to the PG_binding_1 domain from the endolysin of phage SPO1 [61]. To date, there are no other studies confirming the binding capabilities of the DUF5776 domain. DUF3597, another domain with putative peptidoglycan-binding activity, has not been described. DUF5776 and DUF3597 domains were found in 6 and 35 N-acetylmuramoyl-L-alanine amidases of types 3 and 2, respectively. This group of CBDs requires further in-depth study.

#### 4.5.5. The SPOR Domain

The last type of CBD found in this work was SPOR (sporulation-related repeat). SPOR domains are capable of binding to peptidoglycan septa including those formed during sporulation—hence the name of the domain. SPOR domains are rare in bacteria, and their distribution is associated with the structural features of peptidoglycans, namely, the presence or absence of denuded glycans. Bacilli possess proteins with SPOR domains, suggesting that such domains can also be found in the endolysins of *Bacillus*-infecting phages. The domain has been found in the endolysins of phages infecting other bacterial species such as, for example, type 3 N-acetylmuramoyl-L-alanine amidase—PlyGspY412 of the *Geobacillus* sp. Y412MC61 prophage [66,67].

In our bioinformatics analysis, SPOR domains were identified in five endolysins of *Bacillus*-infecting bacteriophages: one of them in a type 2 N-acetylmuramoyl-L-alanine amidase in combination with two SH3 domains, and the other four in type 3 N-acetylmuramoyl-L-alanine amidases as single CBDs. These endolysins have not been experimentally characterized.

Thus, of the seven groups of CBDs found in this study, two (DUF3597 and DUF5776) are of the uncharacterized type, and the remaining have mainly been studied as components of bacterial enzymes. The CBD_PlyG and SH3 domains are the best-studied CBDs in the endolysins of *Bacillus* phages. There is a lack of information on the functional activity and interaction of these domains in their various combinations. However, test systems based on these domains are already in development, indicating their potential for practical application.

The modification of endolysin domains can significantly enhance their properties, increasing their potential against pathogenic bacteria. For example, point amino acid substitutions in endolysin LysAB2, which lyses *A. baumannii*, not only increased its bacteriolytic activity, but also led to a decrease in its hemolytic activity [68]. Removing the CBD of endolysin CD27L against *Clostridium difficile* increased the enzyme’s activity and widened its specificity range [69]. Thus, understanding the domain structure of endolysins is key to developing new antimicrobial molecules. The systematization of information on already characterized enzymes and the creation of a database of endolysins will make it possible to identify enzymes and domains that require more detailed study.

## 5. Conclusions

In this work, 438 genomes of *Bacillus*-infecting bacteriophages were analyzed using bioinformatic methods, leading to the identification of 454 genes encoding endolysins. The domain architecture of all of these endolysins was determined, revealing eight types of EADs and seven types of CBDs. All of the endolysins contained one N-terminal catalytic domain. Two hundred and sixty-eight endolysins contained one EAD and one CBD, ninety-two had one EAD and two CBDs, and nineteen had one EAD and three CBDs. Seventy-five endolysins lacked CBDs, which is atypical for endolysins targeting Gram-positive bacteria including *Bacillus* genus. This could suggest that the CBDs in these endolysins are of types not yet identified or not included in the databases searched in this study.

Phylogenetic trees were constructed for endolysins with the same type of EADs, based on the amino acid sequences of their EADs, except for two groups containing three or fewer sequences: NLPC_P60 domain-containing and MLTF-like domain-containing endolysins. The grouping of the EADs in the trees correlated with both the CBD types and bacterial hosts. Publications on the experimentally characterized endolysins of *Bacillus*-infecting bacteriophages were analyzed, which revealed endolysins that have not been studied experimentally. Endolysins with EAD types of Amidase_2, Amidase_3, and Gly-co_hydro_25 were significantly characterized. At the same time, endolysins with other five types of EADs—Peptidase_M15_3, NlpC_P60, Bacteriophage_GH24, Glycosyl_Hydrolase_73 and MLTF-like—require more detailed study. Significantly fewer studies have been published that have focused on CBDs compared to EADs. Since CBDs are responsible for attachment to the cell wall, they directly affect the specificity of endolysins.

## Data Availability

All data used in this study are included in the article and presented within the text and Supplementary Materials. No further links or repositories are required.

## Supplementary Materials

The following supporting information can be downloaded at: https://www.mdpi.com/article/10.3390/biom14121586/s1, Table S1: Endolysin sequences from *Bacillus*-infecting bacteriophages used in this study.
