# Supplementary material for: Diversity of Endolysin Domain Architectures in Bacteriophages Infecting Bacilli
Source: Biomolecules. 2024 Dec 11;14(12):1586. doi: 10.3390/biom14121586 (PMC11674121; doi:10.3390/biom14121586)
Supplement: Supplementary file 1 [file biomolecules-14-01586-s001.zip › biomolecules-3335695-supplementary.pdf]

biomolecules

MDPI

Supplementary Materials

Diversity of Endolysin Domain Architectures in Bacteriophages Infecting Bacilli

Olga N. Koposova, Olesya A. Kazantseva and Andrey M. Shadrin *

Laboratory of Bacteriophage Biology, G.K. Skryabin Institute of Biochemistry and Physiology of Microorganisms, Pushchino Scientific Center for Biological Research of the Russian Academy of Sciences, Federal Research Center, Prospect Nauki, 5, 142290 Pushchino, Russia; koposova@pbcras.ru (O.N.K.); olesyakazantseva@bk.ru (O.A.K.)
* Correspondence: a.shadrin@pbcras.ru

Table S1. Endolysins sequences from *Bacillus*-infecting bacteriophages used in this study.

№	Genome accession number	Name	EAD	CBD	Protein accession number
		N-acetylmuramoyl-L-alanine amidase type 2			
1	HM144387.1	*Bacillus* phage W.Ph.	Amidase_2	SH3	YP_004957016.1
2	JN654439.1	*Bacillus* phage BPS13	Amidase_2	SH3	YP_006907567.1
3	KC430106.1	*Bacillus* phage BPS10C	Amidase_2	SH3	YP_009002894.1
4	KJ489399.1	*Bacillus* phage Hakuna	Amidase_2	SH3	YP_009036516.1
5	KJ489401.1	*Bacillus* phage Megatron	Amidase_2	SH3	YP_009036139.1
6	KT207918.1	*Bacillus* phage Eyuki	Amidase_2	SH3	ALA46732.1
7	KU737344.1	*Bacillus* phage Nigalana	Amidase_2	SH3	YP_009282466.1
8	KU737350.1	*Bacillus* phage SageFayge	Amidase_2	SH3	AMW62993.1
9	KU737351.1	*Bacillus* phage NotTheCreek	Amidase_2	SH3	AMW63292.1
10	KU737352.1	*Bacillus* phage Nemo	Amidase_2	SH3	AMW63551.1
11	KU847400.1	*Bacillus* phage Crookii	Amidase_2	SH3	1157-1990 bp*
12	KX011169.1	*Bacillus* phage SalinJah	Amidase_2	SH3	YP_009282023.1
13	KX147229.1	*Bacillus* phage Belinda	Amidase_2	SH3	YP_009280443.1
14	KX156152.1	*Bacillus* phage Smudge	Amidase_2	SH3	ANI24688.1
15	KX349902.1	*Bacillus* phage Kida	Amidase_2	SH3	ANU80030.1
16	KX349903.1	*Bacillus* phage DirtyBetty	Amidase_2	SH3	ANT41384.1
17	KX961632.1	*Bacillus* phage SBP8a	Amidase_2	SH3	AOZ62319.1
18	MF288917.1	*Bacillus* phage PPIsBest	Amidase_2	SH3	ASR78311.1
19	MF288918.1	*Bacillus* phage Bubs	Amidase_2	SH3	ASR78719.1
20	MF288920.1	*Bacillus* phage Zainny	Amidase_2	SH3	ASR79286.1
21	MH638311.1	*Bacillus* phage OmnioDeoPrimus	Amidase_2	SH3	AXQ67568.1
22	MN038176.1	*Bacillus* phage Phireball	Amidase_2	SH3	QDH49347.1
23	MN038179.1	*Bacillus* phage ALPS	Amidase_2	SH3	QDH50054.1
24	MT584805.1	*Bacillus* phage Tomato	Amidase_2	SH3	QLF85889.1
25	MW392803.1	*Bacillus* phage BCPG1	Amidase_2	SH3	QQO38733.1
26	MW584228.1	*Bacillus* phage BCPG3	Amidase_2	SH3	QSJ04346.1
27	MW596414.1	*Bacillus* phage BCP18	Amidase_2	SH3	QSJ04559.1
28	OK500001.1	*Bacillus* phage vB_BanH_Abinadi	Amidase_2	SH3	UGO46610.1
29	OM350012.1	*Bacillus* phage Darren	Amidase_2	SH3	ULF48983.1

30	OM350013.1	*Bacillus* phage MrBubbles	Amidase_2	SH3	ULF49279.1
31	KU665491.1	*Bacillus* phage Mgbh1	Amidase_2	2xPG_binding_1	YP_009595180.1
32	OM236514.1	*Bacillus* phage rho14	Amidase_2	LysM	UNY48490.1
33	KC847113.1	*Bacillus* phage PBP180	Amidase_2	PG_binding_1	AGK88055.1
34	JQ062992.1	*Bacillus* phage phIS3501	Amidase_2	SH3	37632-38273 bp*
35	KM236245.1	*Bacillus* phage Mater	Amidase_2	PG_binding_1	YP_009151161.1
36	JX238501.3	*Bacillus* phage phiAGATE	Amidase_2	-	YP_007349224.1
37	KJ010547.1	*Bacillus* phage Bp8p-C	Amidase_2	DUF3597	YP_009227062.1
38	KJ010548.1	*Bacillus* phage Bp8p-T	Amidase_2	DUF3597	AHJ87796.1
39	KM051843.1	*Bacillus* phage Bobb	Amidase_2	-	YP_009056487.1
40	MH707429.1	*Bacillus* phage BSP12	Amidase_2	DUF3597	AYJ76223.1
41	MH707431.1	*Bacillus* phage BSP14	Amidase_2	DUF3597	AYJ75913.1
42	MZ501260.1	*Bacillus* phage 010DV004	Amidase_2	DUF3597	QZA69164.1
43	MZ501261.1	*Bacillus* phage 010DV005	Amidase_2	DUF3597	QZA69444.1
44	MZ501263.1	*Bacillus* phage 043JT007	Amidase_2	DUF3597	QZA70014.1
45	MZ501265.1	*Bacillus* phage 278BB001	Amidase_2	-	QZA70372.1
46	OK349510.1	*Bacillus* phage BM-P1	Amidase_2	2xDUF3597	UJJ74843.1
47	OM728300.1	*Bacillus* phage vB_BsuM-Goe17	Amidase_2	DUF3597	WCS68908.1
48	OM728301.1	*Bacillus* phage vB_BsuM-Goe20	Amidase_2	DUF3598	WCS69164.1
49	OM728303.1	*Bacillus* phage vB_BsuM-Goe25	Amidase_2	DUF3599	WCS69675.1
50	MT151604.1	*Bacillus* phage P59	Amidase_2	-	QIW88648.1
51	MG727696.1	*Paenibacillus* phage Kiel007	Amidase_2	-	AUS03644.1
52	MG727698.1	*Paenibacillus* phage PBL1c	Amidase_2	-	YP_009836351.1
53	MG727699.1	*Paenibacillus* phage Pagassa	Amidase_2	-	YP_009836430.1
54	MG727700.1	*Paenibacillus* phage Tadhana	Amidase_2	-	YP_009836500.1
55	MG727701.1	*Paenibacillus* phage Leyra	Amidase_2	-	AUS03845.1
56	MG727702.1	*Paenibacillus* phage Likha	Amidase_2	-	YP_009836640.1
57	OP066531.2	*Bacillus* phage B13	Amidase_2	2xSH3	UUW40205.1
58	ON528935.2	*Bacillus* phage vB_BteM-A9Y	Amidase_2	LysM; PG_binding_1	USL85042.1
59	DQ150593.1	*Bacillus* phage Fah	Amidase_2	CBD_PlyG	ABA42708.1
60	DQ222851.1	*Bacillus anthracis* phage Cherry	Amidase_2	CBD_PlyG	ABA46392.1
61	DQ222853.1	*Bacillus anthracis* phage (Gamma isolate 51)	Amidase_2	CBD_PlyG	ABA46449.1
62	DQ289555.1	*Bacillus* phage Wbeta	Amidase_2	CBD_PlyG	ABC40416.1
63	DQ289556.1	*Bacillus anthracis* phage (Gamma isolate d'Herelle)	Amidase_2	CBD_PlyG	ABC40469.1
64	EU874396.1	*Bacillus* phage IEBH	Amidase_2	CBD_PlyG	YP_002154393.1
65	GU229986.1	*Bacillus* phage 250	Amidase_2	CBD_PlyG	ADB28383.1
66	HM072038.1	*Bacillus* phage phi105	Amidase_2	LysM	NP_690779.1
67	KF296718.1	*Bacillus* phage phiCM3	Amidase_2	SH3	YP_009009166.1
68	KJ920400.1	*Bacillus* phage Waukesha92	Amidase_2	2xSH3	YP_009099314.1
69	KT970645.1	*Bacillus* phage phi4J1	Amidase_2	-	YP_009218153.1
70	KT970646.1	*Bacillus* phage phiS58	Amidase_2	2xSH3	ALO79948.1
71	MG584725.1	*Bacillus* phage BVE2	Amidase_2	CBD_PlyG	AUG88606.1
72	MK085976.1	*Bacillus* phage AP631	Amidase_2	CBD_PlyG	YP_010739459.1

73	MK843319.1	*Bacillus* phage vB_BthS-TP21T	Amidase_2	2xSH3	QCW20859.1
74	MN065183.1	*Bacillus* phage vB_BthS-HD29phi	Amidase_2	SH3	QDP43493.1
75	OQ317942.1	*Bacillus* phage 0105phi7-2	Amidase_2	CBD_PlyG	YP_010742694.1
76	EU887664.1	*Bacillus* phage TP21-L	Amidase_2	SH3	YP_002333580.1
77	JX887877.1	*Bacillus* phage vB_BtS_BMBtp2	Amidase_2	SH3	YP_007236362.1
78	KF296717.1	*Bacillus* phage proCM3	Amidase_2	SH3	AGV99421.1
79	KT725776.1	*Bacillus* phage vB_BceS-MY192	Amidase_2	CBD_PlyG	ALV83514.1
80	KX190834.1	*Bacillus* phage BMBtpLA3	Amidase_2	SH3	ANT40094.1
81	KX227757.1	*Bacillus* phage PfEFR-4	Amidase_2	CBD_PlyG	ANT40164.1
82	KX227760.1	*Bacillus* phage PfEFR-5	Amidase_2	CBD_PlyG	YP_009285268.1
83	MT136606.1	*Bacillus* phage vB_BceS_KLEB30-3S	Amidase_2	SH3	QIQ68022.1
84	OQ259509.1	*Bacillus* phage BC-7	Amidase_2	CBD_PlyG	WDS60608.1
85	MH884508.1	*Bacillus* phage vB_BcoS-136	Amidase_2	2xSH3; SPOR	YP_010681515.1
86	AP013029.1	*Bacillus* phage phiNIT1	Amidase_2	DUF3597	YP_008318305.1
87	KF669652.1	*Bacillus* phage Grass	Amidase_2	DUF3597	YP_008771412.1
88	LT960608.1	*Bacillus* phage SBSphiJ	Amidase_2	DUF3597	30904-32010 bp*
89	MF422185.1	*Bacillus* phage BSP10	Amidase_2	DUF3597	AUO79588.1
90	MG000860.1	*Bacillus* phage BSP9	Amidase_2	DUF3597	ATN94523.1
91	MH707432.1	*Bacillus* phage BSP15	Amidase_2	DUF3597	AYJ74051.1
92	MH707433.1	*Bacillus* phage BSP18	Amidase_2	DUF3597	AYJ75678.1
93	MH707435.1	*Bacillus* phage BSP21	Amidase_2	DUF3597	AYJ75507.1
94	MW354667.1	*Bacillus* phage BSTP3	Amidase_2	DUF3597	QRI44567.1
95	MZ501262.1	*Bacillus* phage 035JT004	Amidase_2	DUF3597	QZA69723.1
96	OM982668.1	*Bacillus* phage SBSphiJ1	Amidase_2	DUF3597	UPI11801.1
97	OM982669.1	*Bacillus* phage SBSphiJ2	Amidase_2	DUF3597	UPI12052.1
98	OM982670.1	*Bacillus* phage SBSphiJ3	Amidase_2	DUF3597	UPI12307.1
99	OM982671.1	*Bacillus* phage SBSphiJ4	Amidase_2	DUF3597	UPI12559.1
100	OM982672.1	*Bacillus* phage SBSphiJ5	Amidase_2	DUF3597	UPI12806.1
101	OM982673.1	*Bacillus* phage SBSphiJ6	Amidase_2	DUF3597	UPI13050.1
102	OM982674.1	*Bacillus* phage SBSphiJ7	Amidase_2	DUF3597	UPI13298.1
103	KT626446.1	*Bacillus* phage phi4B1	Amidase_2	CBD_PlyG	YP_009206322.1
104	AB930182.1	*Bacillus* phage SPG24	Amidase_2	DUF3597	YP_010582027.1
105	MG727695.1	*Paenibacillus* phage BN12	Amidase_2	-	YP_009836278.1
106	MG727697.1	*Paenibacillus* phage Dragolir	Amidase_2	-	YP_010080174.1
107	KC699836.1	*Bacillus* phage SIOphi	Amidase_2	-	YP_009625688.1
108	MW749002.1	*Bacillus* phage vB_BspH_Mawwa	Amidase_2	-	QXN69853.1
109	MW749007.1	*Bacillus* phage vB_BspH_TimeGriffin	Amidase_2	-	QXN70659.1
110	MW419084.1	*Bacillus* phage 000TH008	Amidase_2	-	QQO40639.1
111	KC595512.2	*Bacillus* phage JL	Amidase_2	2xSH3	YP_009215894.1
112	KC595513.2	*Bacillus* phage Shanette	Amidase_2	2xSH3	YP_009216114.1
113	KF554508.2	*Bacillus* phage CP-51	Amidase_2	2xSH3	YP_009099088.1
114	AF020713.1	*Bacillus* phage SPBc2	Amidase_2	2xSH3	NP_046578.1
115	KY030782.1	*Bacillus* phage phi3T	Amidase_2	2xSH3	APD21160.1
116	MT366945.1	*Bacillus* phage phi3Ts	Amidase_2	2xSH3	QNN96613.1

117	MT366946.1	*Bacillus* phage Hyb1phi3Ts-Spbeta	Amidase_2	2xSH3	QNR51515.1
118	MT366947.1	*Bacillus* phage Hyb2phi3Ts-SPbeta	Amidase_2	2xSH3	QNN96798.1
119	MT366948.1	*Bacillus* phage Hyb3phi3Ts-Spbeta	Amidase_2	2xSH3	QNN96986.1
120	MT601273.1	*Bacillus* phage vB_BsuS-Goe12	Amidase_2	2xSH3	QMV48945.1
121	MT601274.1	*Bacillus* phage vB_BsuS-Goe13	Amidase_2	2xSH3	QMV49120.1
122	MZ969646.1	*Bacillus* phage BUCT082	Amidase_2	2xSH3	UAW07864.1
123	ON107264.1	*Bacillus* phage BUCT083	Amidase_2	2xSH3	UOX38295.1
124	OQ921345.1	*Bacillus* phage SPbetaL5	Amidase_2	-	WIT27619.1
125	OQ921346.1	*Bacillus* phage SPbetaL6	Amidase_2	-	WIT27800.1
126	OQ921347.1	*Bacillus* phage SPbetaL7	Amidase_2	-	WIT27986.1
127	OQ921348.1	*Bacillus* phage SPbetaL8	Amidase_2	-	WIT28172.1
128	KY368640.1	*Bacillus* phage vB_BsuM-Goe3	Amidase_2	DUF3597	YP_009832028.1
129	MN043730.1	*Bacillus* phage vB_BveM-Goe7	Amidase_2	DUF3597	QDP43072.1
130	OM728302.1	*Bacillus* phage vB_BsuM-Goe24	Amidase_2	DUF3597	WCS69423.1
131	OM728304.1	*Bacillus* phage vB_BsuM-Goe27	Amidase_2	DUF3597	WCS69927.1
132	KC481682.1	*Bacillus* phage vB_BanS-Tsamsa	Amidase_2	2xSH3	YP_008873459.1
133	MH884509.1	*Bacillus* phage vB_BboS-125	Amidase_2	2xLysM; PG_binding_1	YP_009841799.1
134	MK288021.1	*Bacillus* phage pW2	Amidase_2	2xSH3	YP_010680031.1
135	MT254578.1	*Bacillus* phage Izhevsk	Amidase_2	2xSH3	YP_010680601.1
136	ON548420.1	*Bacillus* phage vB_BanS-Thrax4	Amidase_2	2xSH3	UUV46714.1
137	KU737348.1	*Bacillus* phage Zuko	Amidase_2	SH3	YP_009291648.1
138	KU737349.1	*Bacillus* phage DIGNKC	Amidase_2	SH3	YP_009278090.1
139	KX961629.1	*Bacillus* phage BJ4	Amidase_2	SH3	AOZ61692.1
140	MF288919.1	*Bacillus* phage AaronPhadg-ers	Amidase_2	SH3	ASR79007.1
141	MG763894.1	*Bacillus* phage HonestAbe	Amidase_2	SH3	AUV57709.1
142	MH538193.1	*Bacillus* phage Saddex	Amidase_2	SH3	AXF41890.1
143	OQ259508.1	*Bacillus* phage BC-6	Amidase_2	-	WDS60557.1
144	MG983742.1	*Bacillus* phage Anath	Amidase_2	CBD_PlyG	AVO23035.1
145	KT070867.1	*Bacillus* phage PBC2	Amidase_2	2xSH3	YP_010679375.1
146	MW084976.1	*Bacillus* phage Kirov	Amidase_2	2xSH3	YP_010679485.1
147	OK499972.1	*Bacillus* phage vB_BanS_Chewbecca	Amidase_2	2xSH3	YP_010681322.1
148	OK499987.1	*Bacillus* phage vB_BanS_MrDarsey	Amidase_2	2xSH3	UGO48021.1
149	OK499991.1	*Bacillus* phage vB_BanS_Sophrita	Amidase_2	2xSH3	YP_010679905.1
150	OK499992.1	*Bacillus* phage vB_BanS_Nate	Amidase_2	2xSH3	YP_010680347.1

151	OK499994.1	*Bacillus* phage vB_BanS_Sky-walker	Amidase_2	2xSH3	YP_010680719.1
152	OM654379.1	*Bacillus* phage vB_BauS_KLEB27-1	Amidase_2	PG_binding_1	UNY40620.1
153	ON548417.1	*Bacillus* phage vB_BanS-Thrax1	Amidase_2	2xSH3	UUV45944.1
154	ON548418.1	*Bacillus* phage vB_BanS-Thrax2	Amidase_2	2xSH3	UUV46199.1
155	ON548419.1	*Bacillus* phage vB_BanS-Thrax3	Amidase_2	2xSH3	UUV46447.1
156	ON548421.1	*Bacillus* phage vB_BanS-Thrax5	Amidase_2	-	UUV46982.1
157	MT601272.1	*Bacillus* phage vB_BsuS-Goe11	Amidase_2	2xSH3	QMV48773.1
158	OL580764.1	*Bacillus* phage vB_BsuS-Goe14	Amidase_2	2xSH3	UIS26467.1
159	OQ921344.1	*Bacillus* phage SPbetaL4	Amidase_2	-	WIT27422.1
160	OM236515.1	*Bacillus* phage SPR	Amidase_2	2xSH3	UNY48566.1
161	ON210834.1	*Bacillus* phage 268TH007	Amidase_2	LysM	URO01719.1
162	ON210835.1	*Bacillus* phage 268TH002	Amidase_2	LysM	URO01821.1
163	OQ921341.1	*Bacillus* phage SPbetaL1	Amidase_2	-	WIT26861.1
164	OQ921342.1	*Bacillus* phage SPbetaL2	Amidase_2	2xSH3	WIT27056.1
165	OQ921343.1	*Bacillus* phage SPbetaL3	Amidase_2	2xSH3	WIT27245.1
166	MH458951.1	*Bacillus* phage vB_BthS_BMBphi	Amidase_2	-	AXF39889.1
167	OQ870554.1	*Bacillus* phage PSYJ-YH	Amidase_2	CBD_PlyG	WKV24081.1
168	KU160496.1	*Bacillus* phage vB_BhaS-171	Amidase_2	-	YP_009273353.1
169	MH606185.1	*Bacillus* phage BSP38	Amidase_2	DUF3597	YP_009840516.1
170	MH707436.1	*Bacillus* phage BSP36	Amidase_2	DUF3597	AYJ75134.1
171	MF418016.1	*Bacillus* phage vB_BceM-HSE3	Amidase_2	2xSH3	AWD93052.1
172	MH884514.1	*Bacillus* phage vB_BpsM-61	Amidase_2	LysM; PG_binding_1	AYP68841.1
173	KT895374.1	*Bacillus* phage vB_BpuM-BpSp	Amidase_2	3xPG_bind-ing_1	ALN97698.1
174	OP380492.1	*Bacillus* phage Nachito	Amidase_2	-	UXR28858.1
175	MT416612.2	*Bacillus* phage YungSlug	Amidase_2	SH3	QKE56405.1
176	KJ024807.2	*Bacillus* phage vB_BtS_BMBtp3	Amidase_2	SH3	YP_009193999.2
177	KT372714.1	*Bacillus* phage vB_BtS_BMBtp16	Amidase_2	SH3	ALF01604.1
178	KT852578.1	*Bacillus* phage BMBtp1	Amidase_2	SH3	ALJ98013.1
179	KX190835.1	*Bacillus* phage vB_BtS_BMBtp15	Amidase_2	SH3	ANT40136.1
180	KX227758.1	*Bacillus* phage PfNC7401	Amidase_2	-	ANT40257.1
181	KX227759.1	*Bacillus* phage PfIS075	Amidase_2	-	ANT40327.1
182	KY963369.1	*Bacillus* phage Tavor_SA	Amidase_2	SH3	YP_010739854.1
183	KY963370.1	*Bacillus* phage Negev_SA	Amidase_2	SH3	YP_010739675.1
184	KY963371.1	*Bacillus* phage Carmel_SA	Amidase_2	SH3	YP_010739566.1
185	MT745954.2	*Bacillus* phage F16Ba	Amidase_2	-	YP_010739622.1
186	MT745955.1	*Bacillus* phage J5a	Amidase_2	-	YP_010739914.1

187	MT745956.2	*Bacillus* phage z1a	Amidase_2	-	YP_010739977.1
188	NC 004820.1	*Bacillus* prophage phBC6A51	Amidase_2	-	NP_852545.1
189	OK499979.1	*Bacillus* Phage vB_BanS_McSteamy	Amidase_2	-	YP_010739733.1
190	OK499999.1	*Bacillus* phage vB_BanS_Booya	Amidase_2	-	YP_010739792.1
191	KU878088.1	*Bacillus* phage AR9	Amidase_2	3xPG_binding_1	YP_009283176.1
192	LC680884.1	*Bacillus* phage PBS1	Amidase_2	3xPG_binding_1	YP_009664366.1
193	MW749003.1	*Bacillus* phage vB_BspM_Internexus	Amidase_2	3xPG_binding_1	QXN70195.1
		N-acetylmuramoyl-L-alanine amidase type 3			
194	MG784342.1	*Bacillus* phage Carmen17	Amidase_3	-	YP_009837328.1
195	MH598512.1	*Bacillus* phage Wes44	Amidase_3	-	YP_009840448.1
196	MZ089978.1	*Bacillus* phage Sato	Amidase_3	SPOR	YP_010771370.1
197	AY616446.1	*Bacillus* phage BCJA1c	Amidase_3	PG_binding_1	YP_164437.1
198	DQ840344.2	*Bacillus* phage 1	Amidase_3	SPOR	YP_001425618.1
199	MN604698.1	*Bacillus* phage vB_BcM_Sam46	Amidase_3	-	QIQ61229.1
200	MN524844.1	*Bacillus* phage vB_Bpu_PumA1	Amidase_3	LysM	YP_009910591.1
201	MN524845.1	*Bacillus* phage vB_Bpu_PumA2	Amidase_3	LysM	YP_009910619.1
202	MW419775.2	*Bacillus* phage WhyPhy	Amidase_3	LysM	YP_010114679.1
203	MH707434.1	*Bacillus* phage BSP19	Amidase_3	LysM	AYJ75628.1
204	MW354669.1	*Bacillus* phage BSTP5	Amidase_3	LysM	QQO90082.1
205	MW354676.1	*Bacillus* phage BSTP8	Amidase_3	LysM	QRI44371.1
206	MW354677.1	*Bacillus* phage BSTP10	Amidase_3	LysM	QRI44396.1
207	MW354678.1	*Bacillus* phage BSTP12	Amidase_3	LysM	QRI44526.1
208	MH707430.1	*Bacillus* phage BSP7	Amidase_3	LysM	AYJ76152.1
209	JN638751.1	*Bacillus* phage G	Amidase_3	LysM	YP_009015334.1
210	KC330679.1	*Bacillus* phage Curly	Amidase_3	2xLysM	YP_007517576.1
211	KC330680.1	*Bacillus* phage Eoghan	Amidase_3	2xLysM	YP_007517421.1
212	KC330681.1	*Bacillus* phage Gemini	Amidase_3	2xLysM	AGE60871.1
213	KC330682.1	*Bacillus* phage Taylor	Amidase_3	2xLysM	AGE60949.1
214	KC330683.1	*Bacillus* phage Finn	Amidase_3	2xLysM	YP_007517651.1
215	KC330684.1	*Bacillus* phage Andromeda	Amidase_3	2xLysM	YP_007517497.1
216	KF669648.1	*Bacillus* phage Blastoid	Amidase_3	2xLysM	YP_008771857.1
217	KF669651.1	*Bacillus* phage Glittering	Amidase_3	2xLysM	YP_008770668.1
218	KF669659.1	*Bacillus* phage Riggi	Amidase_3	2xLysM	YP_008770590.1
219	KU836751.1	*Bacillus* phage Leo2	Amidase_3	2xLysM	AMR60071.1
220	MT422786.1	*Bacillus* phage Novomoskovsk	Amidase_3	2xLysM	QKN88223.1
221	MT514532.1	*Bacillus* phage Bolokhovo	Amidase_3	2xLysM	QMS41902.1
222	MN082625.1	*Bacillus* phage Karezi	Amidase_3	LysM	YP_009910559.1
223	MN857617.1	*Bacillus* phage SRT01hs	Amidase_3	LysM	YP_009910636.1
224	OP433492.1	*Bacillus* phage vB_BaeroP_SYYB1	Amidase_3	LysM	UXN78511.1
225	X96987.2	*Bacteriophage* GA-1 complete	Amidase_3	LysM	NP_073698.1
226	JN712910.1	*Bacillus* phage BCD7	Amidase_3	SPOR	YP_007005928.1

227	AB711120.1	*Bacillus* phage PM1	Amidase_3	DUF5776	YP_007678107.1
228	KU640380.1	*Bacillus* phage Shbh1	Amidase_3	PG_binding_1	YP_009275265.1
229	ON366410.1	*Bacillus* phage vB_BceS_LY1	Amidase_3	-	USL89266.1
230	KP063903.1	*Bacillus* phage BalMu-1 copy2	Amidase_3	2xLysM	AJA42434.1
231	CP027117.1	*Bacillus* phage EZ-2018a	Amidase_3	-	13520-14347 bp*
232	MN176219.1	*Bacillus* phage 000TH010	Amidase_3	DUF5776	YP_010644344.1
233	MN176227.1	*Bacillus* phage 049ML001	Amidase_3	DUF5776	YP_010644428.1
234	MN176228.1	*Bacillus* phage 049ML003	Amidase_3	DUF5776	QFR56412.1
235	X97918.2	Bacteriophage SPP1	Amidase_3	DUF5776	NP_690702.1
236	MN176220.1	*Bacillus* phage 019DV002	Amidase_3	2xLysM	QFG05175.1
237	MN176221.1	*Bacillus* phage 019DV004	Amidase_3	2xLysM	QFG05260.1
238	MN176230.1	*Bacillus* phage 056SW001B	Amidase_3	2xLysM	QFR56498.1
239	OM112210.1	*Bacillus* phage PK2	Amidase_3	LysM	UKL30017.1
240	KC595511.2	*Bacillus* phage Basilisk	Amidase_3	CBD_PlyG	YP_010656823.1
241	MG967616.1	*Bacillus* phage v_B-Bak1	Amidase_3	CBD_PlyG	AXY83006.1
242	MG967617.1	*Bacillus* phage v_B-Bak6	Amidase_3	CBD_PlyG	AXY83126.1
243	MG967618.1	*Bacillus* phage v_B-Bak10	Amidase_3	CBD_PlyG	AXY83225.1
244	JQ619704.1	*Bacillus* phage PBC1	Amidase_3	CBD_PlyG	YP_006383478.1
245	MW392801.1	*Bacillus* phage BCP6	Amidase_3	-	QSJ04276.1
246	MW392802.1	*Bacillus* phage BCPST	Amidase_3	-	YP_010657319.1
247	JN797797.1	*Bacillus* phage BCP78	Amidase_3	SH3	YP_006907849.1
248	KJ676859.1	*Bacillus* phage JBP901	Amidase_3	SH3	YP_009149049.1
249	KT187252.1	*Bacillus* phage PBC6	Amidase_3	SH3	ALA07606.1
250	KT224359.1	*Bacillus* phage TsarBomba	Amidase_3	SH3	YP_009206875.1
251	KT995480.1	*Bacillus* phage Bm15	Amidase_3	SH3	YP_009626617.1
252	KX495186.1	*Bacillus* phage PK16	Amidase_3	SH3	ANY29213.1
253	KX961630.1	*Bacillus* phage QCM8	Amidase_3	SH3	AOZ61963.1
254	KX987999.1	*Bacillus* phage BCP12	Amidase_3	SH3	AQN32617.1
255	MG602477.1	*Bacillus* phage BCP01	Amidase_3	SH3	AUM58831.1
256	MH487649.1	*Bacillus* phage BC01	Amidase_3	SH3	AXU41111.1
257	MH638309.1	*Bacillus* phage Hobo	Amidase_3	SH3	AXQ66832.1
258	MN013089.1	*Bacillus* phage vB_BspM_MarvelLand	Amidase_3	SH3	QEG13565.1
259	MN150686.1	*Bacillus* phage BC-T25	Amidase_3	SH3	QEG04126.1
260	OK499977.1	*Bacillus* phage vB_BanH_Emiliahah	Amidase_3	SH3	UGO49136.1
261	OK499983.1	*Bacillus* phage vB_BanH_McCartney	Amidase_3	SH3	UGO47622.1
262	OL964058.1	*Bacillus* phage vB_BtM_BMBsp2	Amidase_3	SH3	UJH95763.1
263	KP063902.1	*Bacillus* phage BalMu-1 copy 1	Amidase_3	2xLysM	AJA42490.1
264	MN604230.1	*Bacillus* phage vB_BcM_Sam112	Amidase_3	-	QGF21730.1
265	KX190833.1	*Bacillus* phage vB_BtS_BMBtp14	Amidase_3	CBD_PlyG	ANT40035.1
266	MZ089979.1	*Bacillus* phage Sole	Amidase_3	-	QWE49682.1
267	KT001912.1	*Bacillus* phage Silence	Amidase_3	SH3	AKU43363.1
268	MH884513.1	*Bacillus* phage vB_BpsS-36	Amidase_3	SH3	AYP68733.1
269	KT070866.1	*Bacillus* phage PBC4	Amidase_3	CBD_PlyG	YP_010657049.1
270	MK288022.1	*Bacillus* phage pW4	Amidase_3	CBD_PlyG	YP_010657195.1

271	OQ436521.1	*Bacillus* phage BSG01	Amidase_3	CBD_PlyG	WEM05690.1
272	LC597490.1	*Bacillus* phage vB_BceM_WH1	Amidase_3	DUF5776	BCO16118.1
273	JX094431.1	*Bacillus* phage vB_BceM_Bc431v3	Amidase_3	SH3	YP_007676909.1
274	AY701338.1	*Bacillus* phage GIL16c	Amidase_3	SPOR	YP_224129.1
275	EU408779.1	*Bacillus* phage AP50	Amidase_3	CBD_PlyG	YP_002302543.1
276	OP646176.1	*Bacillus* phage vB_BceS_LY5	Amidase_3	CBD_PlyG	UZT28580.1
		Glycosyl hydrolase family 24			
277	ON366412.2	*Bacillus* phage vB_BceP_LY3	Bacterio-phage_GH24	-	USL89550.1
278	KT780304.1	*Bacillus* phage VMY22	Bacterio-phage_GH24	SH3	YP_009198025.1
279	KY821088.1	*Bacillus* phage Harambe	Bacterio-phage_GH24	SH3	YP_009910172.1
280	KY921761.1	*Bacillus* phage BeachBum	Bacterio-phage_GH24	SH3	YP_009910202.1
281	KC685370.1	*Bacillus* phage MG-B1	Bacterio-phage_GH24	SH3	YP_008060123.1
282	MW749009.1	*Bacillus* phage vB_BspP_Dartukuta	Bacterio-phage_GH24	-	QXN70915.1
283	EU622808.1	*Bacillus* phage Nf	Bacterio-phage_GH24	2xLysM	YP_009910732.1
284	EU771092.1	*Bacillus* phage phi29	Bacterio-phage_GH24	2xLysM	YP_002004544.1
285	KU831549.1	*Bacillus* phage vB_BsuP-Goe1	Bacterio-phage_GH24	2xLysM	YP_009910708.1
286	M11813.1	Bacteriophage PZA	Bacterio-phage_GH24	2xLysM	NP_040733.1
287	MF407276.1	*Bacillus* phage vB_BveP-Goe6	Bacterio-phage_GH24	2xLysM	YP_009910346.1
288	MH707426.1	*Bacillus* phage BSP2	Bacterio-phage_GH24	2xLysM	AYJ76495.1
289	MH707427.1	*Bacillus* phage BSP4	Bacterio-phage_GH24	2xLysM	AYJ76471.1
290	MH707428.1	*Bacillus* phage BSP11	Bacterio-phage_GH24	2xLysM	AYJ76448.1
291	MT459794.1	*Bacillus* phage Gxv1	Bacterio-phage_GH24	2xLysM	QKN88717.1
292	MW354668.1	*Bacillus* phage BSTP4	Bacterio-phage_GH24	2xLysM	QQO90053.1
293	MW354670.1	*Bacillus* phage BSTP6	Bacterio-phage_GH24	2xLysM	QRD99848.1
294	MW477480.1	*Bacillus* phage Whiting18	Bacterio-phage_GH24	2xLysM	QRD99298.1
295	OL744111.1	*Bacillus* phage Arbo1	Bacterio-phage_GH24	2xLysM	UIS65831.1
296	OM240926.1	*Bacillus* phage vB_BsuP-Goe15	Bacterio-phage_GH24	2xLysM	UMO75892.1
297	OM240927.1	*Bacillus* phage vB_BsuP-Goe18	Bacterio-phage_GH24	2xLysM	UMO75915.1

298	OM240928.1	*Bacillus* phage vB_BsuP-Goe22	Bacterio-phage_GH24	2xLysM	UMO75941.1
299	OM240929.1	*Bacillus* phage vB_BsuP-Goe23	Bacterio-phage_GH24	2xLysM	UMO75964.1
300	OM249958.1	*Bacillus* phage TBA3	Bacterio-phage_GH24	2xLysM	UKM96361.1
301	ON042750.1	*Bacillus* phage Chedec	Bacterio-phage_GH24	2xLysM	UOX39795.1
302	X99260.1	*Bacillus* phage B103	Bacterio-phage_GH24	2xLysM	NP_690649.1
		Glycosyl hydrolase family 25			
303	JF966203.1	*Bacillus* phage Bastille	Glyco_hydro_25	CBD_PlyG	YP_006907558.1
304	KJ489397.1	*Bacillus* phage CAM003	Glyco_hydro_25	CBD_PlyG	AHZ09496.1
305	KJ489398.1	*Bacillus* phage Evoli	Glyco_hydro_25	CBD_PlyG	YP_009035582.1
306	KJ489400.1	*Bacillus* phage Hoody T	Glyco_hydro_25	CBD_PlyG	AHZ10370.1
307	KU737346.1	*Bacillus* phage Vinny	Glyco_hydro_25	CBD_PlyG	AMW61814.1
308	MF288921.1	*Bacillus* phage OTooleKem-ple52	Glyco_hydro_25	CBD_PlyG	ASR79499.1
309	MF288922.1	*Bacillus* phage Janet	Glyco_hydro_25	CBD_PlyG	ASR79739.1
310	MF498901.1	*Bacillus* phage Anthony	Glyco_hydro_25	CBD_PlyG	ASU00910.1
311	MH638310.1	*Bacillus* phage Kamfam	Glyco_hydro_25	CBD_PlyG	AXQ67278.1
312	MK215646.1	*Bacillus* phage vB_BthM-Goe5	Glyco_hydro_25	CBD_PlyG	AZF89162.1
313	ON366411.1	*Bacillus* phage vB_BceH_LY2	Glyco_hydro_25	-	USL89456.1
314	ON699007.1	*Bacillus* phage BC-1	Glyco_hydro_25	-	UTQ79964.1
315	AJ536073.2	*Bacillus* phage pGIL01	Glyco_hydro_25	-	CAD59971.1
316	AY257527.1	*Bacillus* phage Bam35c	Glyco_hydro_25	-	AAP83499.1
317	CP013282.1	*Bacillus* phage pGIL02	Glyco_hydro_25	-	AND28839.1
318	KJ081346.1	*Bacillus* phage BCP8-2	Glyco_hydro_25	SH3	YP_009149599.1
319	KJ451625.1	*Bacillus* phage Bcp1	Glyco_hydro_25	SH3	YP_009031336.1
320	MF176161.1	*Bacillus* phage Deep-Purple	Glyco_hydro_25	-	YP_009833673.1
321	MH638312.1	*Bacillus* phage Kioshi	Glyco_hydro_25	SH3	AXQ67728.1
322	GU233956.1	*Bacillus* phage 11143	Glyco_hydro_25	CBD_PlyG	ADA84953.1
323	HE614282.1	*Bacillus* phage BceA1	Glyco_hydro_25	CBD_PlyG	CCE73858.1
324	JN191664.1	*Bacillus* phage BtCS33	Glyco_hydro_25	CBD_PlyG	AFL46409.1
325	JN797798.1	*Bacillus* phage BCU4	Glyco_hydro_25	SH3	YP_009783379.1
326	KT967075.1	*Bacillus* phage phi4I1	Glyco_hydro_25	CBD_PlyG	ALN97329.1
327	KX190832.1	*Bacillus* phage vB_BtS_BMBtp13	Glyco_hydro_25	CBD_PlyG	ANT39959.1
328	KX349899.2	*Bacillus* phage Aurora	Glyco_hydro_25	CBD_PlyG	YP_009292384.1
329	KX349900.2	*Bacillus* phage Claudi	Glyco_hydro_25	CBD_PlyG	YP_009279606.1
330	KX349901.1	*Bacillus* phage Stitch	Glyco_hydro_25	CBD_PlyG	YP_009281742.1
331	KX961631.1	*Bacillus* phage QCM11	Glyco_hydro_25	CBD_PlyG	YP_009910137.1
332	KY947509.1	*Bacillus* phage SerPounce	Glyco_hydro_25	CBD_PlyG	YP_009910245.1
333	MF156577.1	*Bacillus* phage Juan	Glyco_hydro_25	CBD_PlyG	YP_009910281.1
334	MF156578.1	*Bacillus* phage KonjoTrouble	Glyco_hydro_25	CBD_PlyG	YP_009910321.1
335	MF156580.1	*Bacillus* phage RadRaab	Glyco_hydro_25	CBD_PlyG	ASU04191.1
336	MG710484.1	*Bacillus* phage BtiUFT6.51- F	Glyco_hydro_25	CBD_PlyG	AUO78580.1
337	MH817022.1	*Bacillus* phage vB_BthP-Goe4	Glyco_hydro_25	CBD_PlyG	AYD87743.1
338	MK084630.1	*Bacillus* phage StevenHerd11	Glyco_hydro_25	CBD_PlyG	AZF88338.1
339	MK284526.1	*Bacillus* phage DK1	Glyco_hydro_25	CBD_PlyG	YP_009910435.1

340	MK284527.1	*Bacillus* phage DK2	Glyco_hydro_25	CBD_PlyG	YP_009910482.1
341	MK284528.1	*Bacillus* phage DK3	Glyco_hydro_25	CBD_PlyG	YP_009910529.1
342	MK310228.1	*Bacillus* phage vB_BthP-HD73phi	Glyco_hydro_25	CBD_PlyG	AZV00054.1
343	MK759918.1	*Bacillus* phage vB_BtS_B83	Glyco_hydro_25	CBD_PlyG	QCQ57806.1
344	MN082624.1	*Bacillus* phage VioletteMad	Glyco_hydro_25	CBD_PlyG	QDH50316.1
345	MT777452.1	*Bacillus* phage Baseball field	Glyco_hydro_25	CBD_PlyG	QOC56865.1
346	MW012634.1	*Bacillus* phage DLc1	Glyco_hydro_25	CBD_PlyG	YP_010113770.1
347	MW348917.1	*Bacillus* phage Thornton	Glyco_hydro_25	CBD_PlyG	QQM15026.1
348	MZ384014.1	*Bacillus* phage DLn1	Glyco_hydro_25	CBD_PlyG	QWT50671.1
349	NC 004821.1	*Bacillus* prophage phBC6A52	Glyco_hydro_25	CBD_PlyG	NP_852605.1
350	OK500002.1	*Bacillus* phage vB_BanS_Athena	Glyco_hydro_25	CBD_PlyG	UGO51742.1
351	OL744112.1	*Bacillus* phage Ademby	Glyco_hydro_25	CBD_PlyG	UIS65867.1
352	ON741189.1	*Bacillus* phage vB_BceS_LY4	Glyco_hydro_25	CBD_PlyG	UTQ80142.1
353	OQ187816.1	*Bacillus* phage BC-5	Glyco_hydro_25	SH3	WCR32947.1
354	MH752385.1	*Bacillus* phage Ray17	Glyco_hydro_25	2xLysM	YP_010644211.1
355	MN013088.1	*Bacillus* phage vB_BspS_SplendidRed	Glyco_hydro_25	2xLysM	YP_010644285.1
356	KU878088.1	*Bacillus* phage AR9	Glyco_hydro_25	3xLysM	YP_009283099.1
357	LC680884.1	*Bacillus* phage PBS1	Glyco_hydro_25	3xLysM	YP_009664287.1
358	OM236516.1	*Bacillus* phage FADO	Glyco_hydro_25	SH3; 2xLysM	YP_010740188.1
359	OM634653.1	*Bacillus* phage vB_BsuS_PJN02	Glyco_hydro_25	SH3; 2xLysM	UNH58402.1
360	OM728297.1	*Bacillus* phage vB_BsuM-Goe21	Glyco_hydro_25	3xLysM	WCS68321.1
361	ON133270.1	*Bacillus* phage 2S-4	Glyco_hydro_25	SH3; 2xLysM	UPG35841.1
362	ON881243.1	*Bacillus* phage PK-3	Glyco_hydro_25	SH3; 2xLysM	UUG68082.1
363	MN176231.1	*Bacillus* phage 276BB001	Glyco_hydro_25	2xLysM	QFG05873.1
364	MN176232.1	*Bacillus* phage 280BB001	Glyco_hydro_25	2xLysM	QFG05954.1
365	MW394467.1	*Bacillus* phage 268TH004	Glyco_hydro_25	2xLysM	QQO40378.1
366	MZ501264.1	*Bacillus* phage 274BB002	Glyco_hydro_25	2xLysM	QZA70103.1
367	EF583821.1	*Bacillus* phage 0305phi8-36	Glyco_hydro_25	-	YP_001429728.1
368	KT070868.1	*Bacillus* phage PBC5	Glyco_hydro_25	-	AKQ08598.1
369	MN176226.1	*Bacillus* phage 035JT001	Glyco_hydro_25	3xLysM	QFG05701.1
370	MN176222.1	*Bacillus* phage 022DV001	Glyco_hydro_25	3xLysM	QFG05345.1
371	MN176223.1	*Bacillus* phage 031MP003	Glyco_hydro_25	3xLysM	QFG05433.1
372	MN176224.1	*Bacillus* phage 031MP002	Glyco_hydro_25	3xLysM	QFG05523.1
373	MN176225.1	*Bacillus* phage 031MP004	Glyco_hydro_25	3xLysM	QFG05610.1
374	MN176229.1	*Bacillus* phage 055SW001	Glyco_hydro_25	3xLysM	QFG05784.1
375	KM236245.1	*Bacillus* phage Mater	Glyco_hydro_25	-	YP_009151164.1
376	MT700412.1	*Bacillus* phage 1_ICo-2020	Glyco_hydro_25	CBD_PlyG	QNI20380.1
377	KT895374.1	*Bacillus* phage vB_BpuM-BpSp	Glyco_hydro_25	3xLysM	ALN97934.1
			Glycosyl hydrolase family 73		
378	KF669647.1	*Bacillus* phage BigBertha	Glycosyl_Hydrolase_73	-	YP_008771117.1
379	AJ536073.2	*Bacillus* phage pGIL01	Glycosyl_Hydrolase_73	-	CAD59966.2
380	AY257527.1	*Bacillus* phage Bam35c	Glycosyl_Hydrolase_73	-	AAP83495.1

381	AY701338.1	*Bacillus* phage GIL16c	Glycosyl_Hydrolase_73	-	AAW33590.1
382	CP013282.1	*Bacillus* phage pGIL02	Glycosyl_Hydrolase_73	-	AND28843.1
383	EU408779.1	*Bacillus* phage AP50	Glycosyl_Hydrolase_73	-	ACB54924.1
384	KF188458.1	*Bacillus* phage Wip1	Glycosyl_Hydrolase_73	-	AGT13379.1
385	MZ089978.1	*Bacillus* phage Sato	Glycosyl_Hydrolase_73	-	QWE49648.1
386	MZ089979.1	*Bacillus* phage Sole	Glycosyl_Hydrolase_73	-	QWE49678.1
387	OM112209.1	*Bacillus* phage PK1	Glycosyl_Hydrolase_73	2xLysM	5679-6482 nt
388	KU878088.1	*Bacillus* phage AR9	Glycosyl_Hydrolase_73	-	AMS01110.1
389	LC680884.1	*Bacillus* phage PBS1	Glycosyl_Hydrolase_73	-	BDE75454.1
390	OM728297.1	*Bacillus* phage vB_BsuM-Goe21	Glycosyl_Hydrolase_73	-	WCS68138.1
391	JN638751.1	*Bacillus* phage G	Glycosyl_Hydrolase_73	-	AEO93294.1
392	OK499991.1	*Bacillus* phage vB_BanS_Sophrita	Glycosyl_Hydrolase_73	-	UGO50820.1
			Transglycosylase		
393	MW749006.1	*Bacillus* phage vB_BspM_AgentSmith	MLTF-like	-	QXN70525.1
			NLPC/P60		
394	KC685370.1	*Bacillus* phage MG-B1	NLPC_P60	-	YP_008060121.1
395	KP411017.1	*Bacillus* phage Palmer	NLPC_P60	-	AJK28086.1
396	MH538296.1	*Bacillus* phage Maceta	NLPC_P60	-	AXF42210.1
			Peptidase M15		
397	OM728298.1	*Bacillus* phage vB_BsuM-Goe16	Peptidase_M15C	PG_binding_1	WCS68525.1
398	MW419085.1	*Bacillus* phage 000TH009	Peptidase_M15C	PG_binding_1	QQO40904.1
399	MW419086.1	*Bacillus* phage 015DV002	Peptidase_M15C	PG_binding_1	QQO41156.1
400	MW419087.1	*Bacillus* phage 015DV004	Peptidase_M15C	PG_binding_1	QQO41433.1
401	MH884512.1	*Bacillus* phage vB_BpsS-140	Peptidase_M15C	PG_binding_1	AZS49230.1
402	KU577463.1	*Bacillus* phage Deep	Peptidase_M15C	2xSH3	YP_009285532.1
403	ON975036.1	*Bacillus* phage Thurquoise	Peptidase_M15C	SH3	UXQ88909.1
404	KF669661.1	*Bacillus* phage Slash	Peptidase_M15C	PG_binding_1	AGY48347.1
405	KF669663.1	*Bacillus* phage Staley	Peptidase_M15C	PG_binding_1	YP_008770774.1
406	KP696448.1	*Bacillus* phage Stills	Peptidase_M15C	PG_binding_1	YP_009196941.1
407	KF669655.1	*Bacillus* phage Page	Peptidase_M15C	PG_binding_1	YP_008770531.1
408	KF669660.1	*Bacillus* phage Pony	Peptidase_M15C	PG_binding_1	YP_008771342.1
409	KM236248.1	*Bacillus* phage Pookie	Peptidase_M15C	PG_binding_1	YP_009152823.1
410	KT001911.1	*Bacillus* phage Pavlov	Peptidase_M15C	PG_binding_1	AKQ07445.1
411	FJ230960.1	*Bacillus* phage SPO1	Peptidase_M15C	2xPG_binding_1	YP_002300379.1
412	KF669649.1	*Bacillus* phage CampHawk	Peptidase_M15C	PG_binding_1	AGY46982.1

413	KY368639.1	*Bacillus* phage vB_BsuM-Goe2	Peptidase_M15C	2xPG_binding_1	APZ82341.1
414	MT601270.1	*Bacillus* phage vB_BsuM-Goe9	Peptidase_M15C	2xPG_binding_1	QMV48409.1
415	MT601271.1	*Bacillus* phage vB_BsuM-Goe10	Peptidase_M15C	2xPG_binding_2	QMV48633.1
416	MW001214.1	*Bacillus* phage SP8	Peptidase_M15C	2xPG_binding_2	QPX71670.1
417	MZ751040.1	*Bacillus* phage phi18	Peptidase_M15C	2xPG_binding_3	UAV84373.1
418	OM728299.1	*Bacillus* phage vB_BsuM-Goe19	Peptidase_M15C	2xPG_binding_3	WCS68744.1
419	OQ921336.1	*Bacillus* phage SPO1L1	Peptidase_M15C	PG_binding_1	WIT25841.1
420	OQ921337.1	*Bacillus* phage SPO1L2	Peptidase_M15C	PG_binding_1	WIT26040.1
421	OQ921338.1	*Bacillus* phage SPO1L3	Peptidase_M15C	PG_binding_1	WIT26236.1
422	OQ921339.1	*Bacillus* phage SPO1L4	Peptidase_M15C	PG_binding_1	WIT26437.1
423	OQ921340.1	*Bacillus* phage SPO1L5	Peptidase_M15C	PG_binding_1	WIT26634.1
424	KM236247.1	*Bacillus* phage Pascal	Peptidase_M15C	PG_binding_1	YP_009151490.1
425	KP696447.1	*Bacillus* phage Stahl	Peptidase_M15C	PG_binding_1	YP_009203662.1
426	JN790865.1	*Bacillus* phage B4	Peptidase_M15C	SH3	YP_006908235.1
427	JN797796.1	*Bacillus* phage B5S	Peptidase_M15C	SH3	AEW47240.1
428	KF208639.2	*Bacillus* phage Troll	Peptidase_M15C	SH3	AGT13629.1
429	KF669662.1	*Bacillus* phage Spock	Peptidase_M15C	SH3	YP_008770279.1
430	KJ489402.1	*Bacillus* phage Riley	Peptidase_M15C	SH3	YP_009055819.1
431	KT307976.1	*Bacillus* phage AvesoBmore	Peptidase_M15C	SH3	ALA13301.1
432	KU737345.1	*Bacillus* phage Juglone	Peptidase_M15C	SH3	AMW61488.1
433	KU737347.1	*Bacillus* phage Phrodo	Peptidase_M15C	SH3	AMW62097.1
434	KY888882.1	*Bacillus* phage Flapjack	Peptidase_M15C	SH3	ARQ94970.1
435	MF765814.1	*Bacillus* phage Taffo16	Peptidase_M15C	SH3	ASZ75788.1
436	MN038178.1	*Bacillus* phage Beyonphe	Peptidase_M15C	SH3	QDH49746.1
437	MN153503.1	*Bacillus* phage Chotacabras	Peptidase_M15C	SH3	QEM43108.1
438	MW281503.1	*Bacillus* phage Anthos	Peptidase_M15C	SH3	QPY77291.1
439	MW787012.1	*Bacillus* phage SWEP1	Peptidase_M15C	SH3	QVW28823.1
440	OK499976.1	*Bacillus* phage vB_BanH_Jar-Jar	Peptidase_M15C	SH3	UGO48868.1
441	OK499990.1	*Bacillus* phage vB_BanH_RonSwanson	Peptidase_M15C	SH3	UGO50359.1
442	OM350011.1	*Bacillus* phage BillyBob	Peptidase_M15C	SH3	ULF48676.1
443	OM743306.1	*Bacillus* phage vB_BcgM	Peptidase_M15C	SH3	UNA01464.1
444	KF669647.1	*Bacillus* phage BigBertha	Peptidase_M15C	2xSH3	YP_008771084.1
445	AB605730.1	*Bacillus* phage SP10	Peptidase_M15C	PG_binding_1	YP_007003388.1
446	LT960610.1	*Bacillus* phage SBSphiC	Peptidase_M15C	PG_binding_1	136003-136749
447	OM728296.1	*Bacillus* phage vB_BsuM-Goe26	Peptidase_M15C	PG_binding_1	WCS68002.1
448	KM236246.1	*Bacillus* phage Moonbeam	Peptidase_M15C	PG_binding_1	YP_009151593.1
449	KU253712.1	*Bacillus* phage Eldridge	Peptidase_M15C	PG_binding_1	YP_009274740.1
450	MN043729.1	*Bacillus* phage vB_BmeM-Goe8	Peptidase_M15C	PG_binding_1	YP_009849987.1
451	KT624200.1	*Bacillus* phage SP-15	Peptidase_M15C	2xPG_binding_1	YP_009302456.1
452	KF188458.1	*Bacillus* phage Wip1	Peptidase_M15C	-	AGT13385.1

453	OM236513.1	*Bacillus* phage SP82G	Peptidase_M15C	2xPG_binding_1	UNY49055.1
454	OM634661.1	*Bacillus* phage vB_BauM_KLEB27-3	Peptidase_M15C	PG_binding_1	UNY39879.1

*The localization of the gene encoding endolysin in the phage genome (when a protein accession number is unavailable).
